# Structure determination and crystal chemistry of large repeat mixed-layer hexaferrites

**DOI:** 10.1107/S2052252518011351

**Published:** 2018-09-12

**Authors:** C. Delacotte, G. F. S. Whitehead, M. J. Pitcher, C. M. Robertson, P. M. Sharp, M. S. Dyer, J. Alaria, J. B. Claridge, G. R. Darling, D. R. Allan, G. Winter, M. J. Rosseinsky

**Affiliations:** a Department of Chemistry, University of Liverpool, Liverpool L69 7ZD, UK; b Department of Physics, University of Liverpool, Liverpool L69 7ZE, UK; c Diamond Light Source, Harwell Campus, Chilton, Oxon OX11 0DE, UK

**Keywords:** large repeat hexaferrites, mixed-layer structural models, polytypes, stacking sequences, defects, magnetic properties

## Abstract

Hexaferrites are an important class of magnetic oxides with applications in data storage and electronics. Their crystal structures are highly modular, consisting of Fe- or Ba-rich close-packed blocks that can be stacked in different sequences to form a multitude of unique structures, producing large anisotropic unit cells with lattice parameters typically >100 Å along the stacking axis. This has limited atomic-resolution structure solutions to relatively simple examples such as Ba_2_Zn_2_Fe_12_O_22_, whilst longer stacking sequences have been modelled only in terms of block sequences, with no refinement of individual atomic coordinates or occupancies. This paper describes the growth of a series of complex hexaferrite crystals, their atomic-level structure solution by high-resolution synchrotron X-ray diffraction, electron diffraction and imaging methods, and their physical characterization by magnetometry. The structures include a new hexaferrite stacking sequence, with the longest lattice parameter of any hexaferrite with a fully determined structure.

## Abstract 2

The structures of a family of long repeat mixed-layer hexaferrites are solved with atomic resolution for the first time using synchrotron X-ray diffraction.

## 1. Introduction   

Hexagonal ferrites or so-called hexaferrites, distinct from cubic or spinel ferrites, have been the subject of numerous studies since their discovery in the 1950s and are still extensively investigated for their magnetic and microwave properties (Batlle *et al.*, 1991 ▸; Liu *et al.*, 2006 ▸; Stergiou & Litsardakis, 2016 ▸). These materials present a wide variety of applications and are widely used as magnets, magnetic recording or data storage materials and electrical device components (especially those operating at microwave frequencies for antennas, isolators, filters *etc*.) (Özgür *et al.*, 2009 ▸). The hexaferrites are a family of magnetic iron(III)-based oxides with hexagonal, trigonal or rhombohedral crystal structures related to the Pb[Fe,Mn]_12_O_19_ magnetoplumbite structure type. There are many variations of hexaferrites (Pullar, 2012 ▸), the most common member being the BaFe_12_O_19_ M-type hexaferrite, which is well known for its use as a permanent magnet. Besides M-type, the main hexaferrites are Ba*M*_2_Fe_16_O_27_ (so-called W-type), Ba_2_ *M*_2_Fe_12_O_22_ (Y-type), Ba_3_ *M*_2_Fe_24_O_41_ (Z-type), Ba_2_ *M*_2_Fe_28_O_46_ (X-type) and Ba_4_ *M*_2_Fe_36_O_60_ (U-type), where **M** = Co^2+^, Zn^2+^, Fe^2+^, Mg^2+^, Mn^2+^ *etc*. The best known hexaferrite compounds are those where the divalent *M* cation is cobalt, however, the hexaferrites described herein contain zinc as the divalent *M* cation.

**Figure 1:**
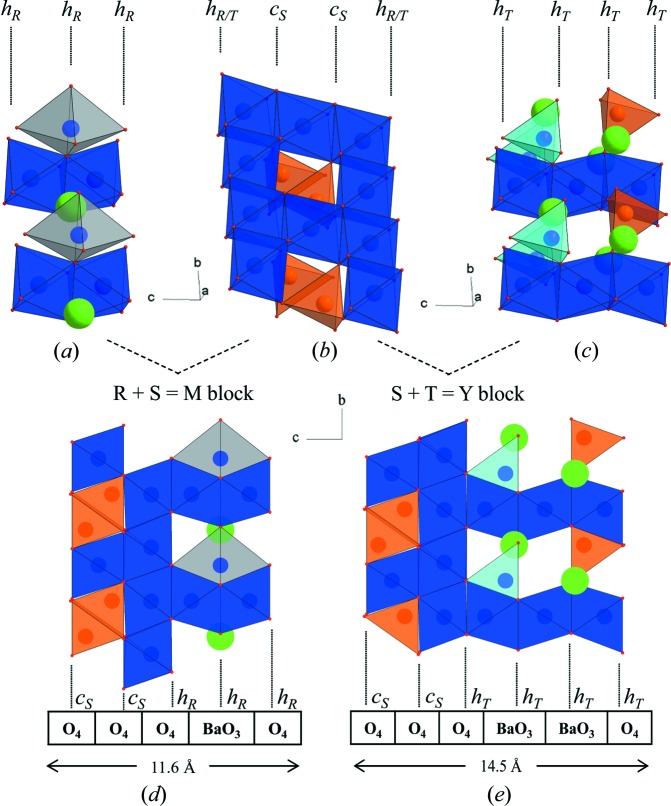
Three-dimensional views of (*a*) the R, (*b*) the S and (*c*) the T sub-blocks and projections of (*d*) the M and (*e*) the Y blocks. Fe^3+^ octahedra are represented in dark blue, Fe^3+^ bipyramids in grey, Fe^3+^ tetrahedra in light blue and mixed Fe^3+^/Zn^2+^ tetrahedra in orange. The *h* and *c* notations indicate hexagonal and cubic packing of the oxygen layers, respectively. Subscript letters correspond to the sub-block types these oxygen layers belong to. The anion-layer stacking and *c* dimension of the M and Y unit blocks are also highlighted.

In terms of physical properties, microwave and mainly magnetic properties have been investigated. All hexaferrites are ferrimagnetic and characterized by high magnetic ordering temperatures owing to the high concentration of Fe^3+^ cations and strong Fe^3+^—O—Fe^3+^ antiferromagnetic superexchange interactions. The crystal structures of these materials can be described with three distinct block types (R, S and T) as described in Fig. 1 ▸ and Section 2. It is possible to rationalize the magnetic moment of different structures by considering the block sequences of these complex structures. The orientation of the magnetic moments associated with the different blocks are opposing but with unequal amplitudes, giving rise to a ferrimagnetic net moment. One of the main characteristics of the magnetic properties of the hexaferrite family is the large magnetocrystalline anisotropy constants, making them attractive as permanent magnets. This means they all present a preferred magnetization orientation that is either uniaxial with the magnetization parallel to the *c* axis in the hexagonal basal plane, or in a cone at an angle to the *c* axis (Özgür *et al.*, 2009 ▸; Pullar, 2012 ▸). In general, there are three main contributions explaining the origin of magnetocrystalline anisotropy: dipole–dipole interactions, spin–orbit coupling and excited states mixed into the ground states. The latter is the dominant mechanism in common hexaferrites presenting uniaxial anisotropy where the presence of a large divalent cation (usually Ba^2+^ or Sr^2+^) causes perturbation in the lattice, creating an unusual fivefold symmetry at the trigonal bipy-ramidal site in the R block. The absence of five-coordinate Fe^3+^ sites in the Y type and the increase of spin–orbit contribution in Co-containing hexaferrites accounts for the observed in-plane and in-cone anisotropy (Pullar, 2012 ▸). Their magnetic properties are thus intrinsically related to their crystal structures. Structural and magnetic characteristics of the main hexaferrites are shown in Table 1 ▸. Recently, magnetically induced magnetoelectric/multiferroic properties have also been observed in some hexaferrites presenting a helical–spiral magnetic structure stabilized by the in-cone anisotropy. Here the magnetoelectric and ferroelectric properties are driven by the breaking of time-reversal symmetry caused by the presence of antisymmetric superexchange in the magnetic structure as opposed to the conventional spatial inversion in polar materials. For instance, the Sr_3_Co_2_Fe_24_O_41_ Z-type hexaferrite was reported to have a field-induced transverse conical magnetic structure at room temperature, resulting in a magnetoelectric effect (Soda *et al.*, 2011 ▸).

## 2. The crystal structure of hexaferrites   

The hexaferrite family contains a subgroup of more complex mixed-layer structures formed from the regular stacking of M and Y unit blocks, the M_*p*_Y_*n*_ series, which was originally incorrectly described as a (TS)_*n*_T series (Kohn & Eckart, 1963 ▸, 1964 ▸). This gives rise to a large number of different stacking sequences with hexagonal *c* parameters up to 1577 Å. In this study, all mixed-layer hexaferrites correspond to a certain arrangement of M and Y blocks (herein ‘mixed layer’ will always refer to MY hexaferrites) within the Ba–Fe–Zn–O system. These M and Y structural unit blocks are themselves built from the three fundamental sub-blocks (the so-called R, S and T blocks, Fig. 1 ▸), which are distinguished by the stacking of their close-packed oxygen layers. In the R and T sub-blocks, the oxygen layers adopt hexagonal close packing, in contrast to cubic close packing in the S sub-blocks. Two types of oxygen layers are present: one with a barium substitution (the {BaO_3_} layers) and one without (the {O_4_} layers).

In the three-layered R block (Fig. 1 ▸ *a*), which is part of the M block, the iron species are in octahedral and bipyramidal environments. Octahedra are connected to each other by sharing faces and share corners with the trigonal bipyramids. The latter are unique positions only found within the R block, corresponding to the shared common face of two tetrahedral sites. This block type is also characterized by the presence of a {BaO_3_} close-packed layer where barium cations are substituted for 25% of the oxide anions. The R block bears a negative formal charge as its composition is [BaFe_6_O_11_]^2−^.

The two-layered S block is a spinel-type block and is found in both M and Y unit blocks. Tetrahedral and octahedral environments are present in this block. Tetrahedra are connected to octahedra by corners, while octahedra are linked with each other by edges (Fig. 1 ▸ *b*). Octahedra are only occupied by Fe^3+^, whereas tetrahedra can be populated by both Fe^3+^ and Zn^2+^ species. Depending on the cation substitution, two types of S blocks can be formed: S^0^ and S^2+^. If all tetrahedral sites are fully occupied by Fe^3+^, the S-block composition is [Fe_6_O_8_]^2+^ and exhibits a positive charge. When Zn^2+^ is substituted, this positive charge then decreases until a Zn_2_Fe_4_O_8_ stoichiometry is reached.

The four-layered T block (Fig. 1 ▸ *c*), which is part of the Y block, consists of face-shared iron octahedra which are linked by corners to tetrahedra. The latter are either iron sites or mixed iron/zinc sites. If all tetrahedral sites are fully occupied by Fe^3+^, the resulting composition is Ba_2_Fe_8_O_14_ and the block is neutral. On the other hand, when Zn^2+^ is substituted, the block becomes negatively charged. The T block also shows the presence of two consecutive {BaO_3_} close-packed layers.

Finally, the stacking of an R and an S block produces an M unit block (Fig. 1 ▸ *d*), while stacking of a T and an S block produces a Y unit block (Fig. 1 ▸ *e*). The M block can thus be described as a 11.6 Å unit with a repeat of five oxygen layers, one of which contains a substituted barium cation. Similarly, the Y block corresponds to a 14.5 Å unit with a repeat of six oxygen layers, two of which (adjacent) contain a substituted barium cation.

**Figure 2:**
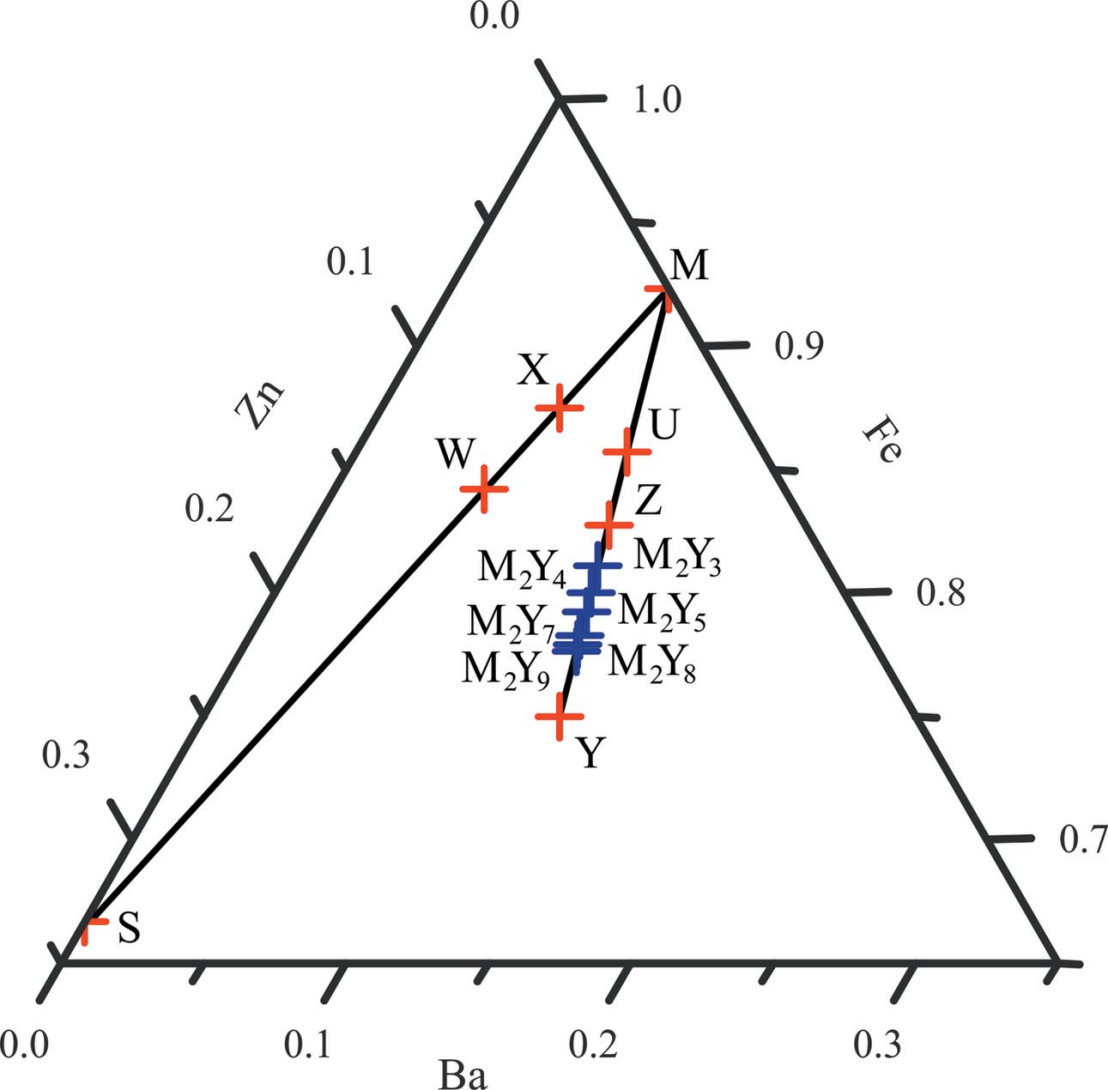
The Ba–Zn–Fe pseudoternary phase diagram showing the M_*p*_S and M_*p*_Y_*n*_ mixed layer subgroups of the hexaferrites. The M_*p*_S subgroup lie on the line between M and the ZnFe_2_O_4_ spinel (S), and the M_*p*_Y_*n*_ subgroup lies on the line between M and Y. The compositions of the hexaferrites with previously reported crystal structures (Table 2 ▸) are shown in red, and those of the hexaferrites with crystal structures reported in this work (Table 4 ▸) are shown in blue.

Among the hexaferrite compounds mentioned in Table 1 ▸, only the Z- and U-type belong to the M_*p*_Y_*n*_ mixed-layer subgroup (Table 2 ▸). The W- and X-type hexaferrites belong to a different mixed-layer subgroup, namely the M_*p*_S series (Fig. 2 ▸).

The mixed-layer M_*p*_Y_*n*_ hexaferrite subgroup forms a wide extended system because the M:Y ratio varies over a relatively large range (Table 3 ▸) and phases belonging to that system exhibit polymorphism, particularly polytypism. Indeed, these compounds are characterized by their chemical composition and by the ordering of the M and Y blocks. For one given stacking-sequence length, depending on the possible M/Y permutations, different polytypes can be formed. For instance, the M_2_Y_4_ series may give rise to the (MYMY_3_)_3_, (MMY_4_)_3_ and (MY_2_)_2_ polytypes. Note that the only two mixed-layer compounds reported with a known crystal structure (*i.e.* where refined atomic positions and occupancies are presented) are the Ba_3_Co_2_Fe_24_O_41_ Z-type and Sr_4_Co_2_Fe_36_O_60_ U-type hexaferrites (Pullar, 2012 ▸). In the Ba–Fe–Zn–O system, the only previous report related to a mixed-layer compound is a Mössbauer study of the Z-type hexaferrite, where no refined structure is presented.

In this paper, we focus on mixed-layer hexaferrites (excluding the known Z and U-types) constructed from different ratios and permutations of M and Y blocks. The earliest examples of mixed-layer M_*p*_Y_*n*_ ferrites were the M_2_Y_*n*_ (Kohn & Eckart, 1964 ▸, 1965*a* ▸,*b* ▸) and M_4_Y_*n*_ (Kohn & Eckart, 1967 ▸) series. As summarized in Table 3 ▸, this earlier work has assigned crystal symmetry and stacking sequences depending on the observed cell lengths. The mixed-layer hexaferrites, characterized by large hexagonal *c* axis values are thus reported as compounds with ideal compositions based on M_*p*_Y_*n*_ sequences that have been determined by combined X-ray diffraction and electron microscopy (Kohn & Eckart, 1967 ▸; Cook, 1967 ▸). Specifically, mixed-layer stacking sequences were established by TEM studies of etch-pit replicas and direct lattice imaging (Kohn & Eckart, 1967 ▸; Cook, 1967 ▸; Kohn *et al.*, 1971 ▸; Van Landuyt *et al.*, 1974 ▸; McConnell *et al.*, 1974 ▸). A one-dimensional 00*l* structure-factor calculation was then used to confirm their stacking models but there was no attempt to determine and refine the complete crystal structures with associated determination of atomic positions and site occupancies. The difficulties in collecting and refining structural data on these large cells have so far prevented detailed structural analysis of the most complex materials within the mixed-layer hexaferrite subfamily.

Here we report full structural refinement of several mixed-layer materials belonging to the M_2_Y_3_, M_2_Y_4_/M_4_Y_8_, M_2_Y_5_, M_2_Y_7_, M_2_Y_8_ and M_2_Y_9_ series (Fig. 2 ▸), which were obtained as single crystals of the Ba–Fe–Zn–O system. We determined their structures crystallographically, combining transmission microscopy techniques with single-crystal X-ray diffraction in order to refine complete structural models. In addition to permitting refinement of the structures of several of the previously reported materials, this revealed the existence of a new stacking sequence within the M_4_Y_8_ series with a complex structure that corresponds to the largest repeats known for an oxide material within the ICSD (Inorganic Crystal Structure Database; http://www2.fiz-karlsruhe.de/icsd_home.html). The magnetic properties of one polytype of the M_2_Y_4_ series were determined.

## 3. Synthesis   

Materials were synthesized by solid-state reactions using a single-crystal flux-growth method. Hexaferrite crystals have been grown previously using NaFeO_2_ (Savage & Tauber, 1964 ▸), Na_2_CO_3_ (Gambino & Leonhard, 1961 ▸), BaF_2_ (Brixner, 1959 ▸) or B_2_O_3_ (Savage & Tauber, 1967 ▸) fluxes, and B_2_O_3_ and NaFeO_2_ have been used to obtain the mixed-layer compounds, which were always obtained within a mixture of different phases corresponding to various mixed-layer and common hexaferrites (such as Z-, M- and Y-type) (Savage & Tauber, 1967 ▸). There was no indication of any dependence of crystal type upon the flux used, but it was reported that B_2_O_3_ gave a better crystal quality compared with NaFeO_2_.

Based on ratios reported by Savage & Tauber (1967 ▸), BaCO_3_ (Alfa Aesar 99%), Fe_2_O_3_ (Alfa Aesar 99.998%) and ZnO (Alfa Aesar 99.99%) precursors and B_2_O_3_ (used as flux) were mixed and ground by hand with an overall mass of 1 g. The mixture was then loaded into a platinum crucible and fired in air: the reaction was homogenized by heating at 1290°C for 3 h and then cooled slowly to 1020°C at a rate of 0.2°C min^−1^. The mixture was then furnace cooled to room temperature.

**Figure 3:**
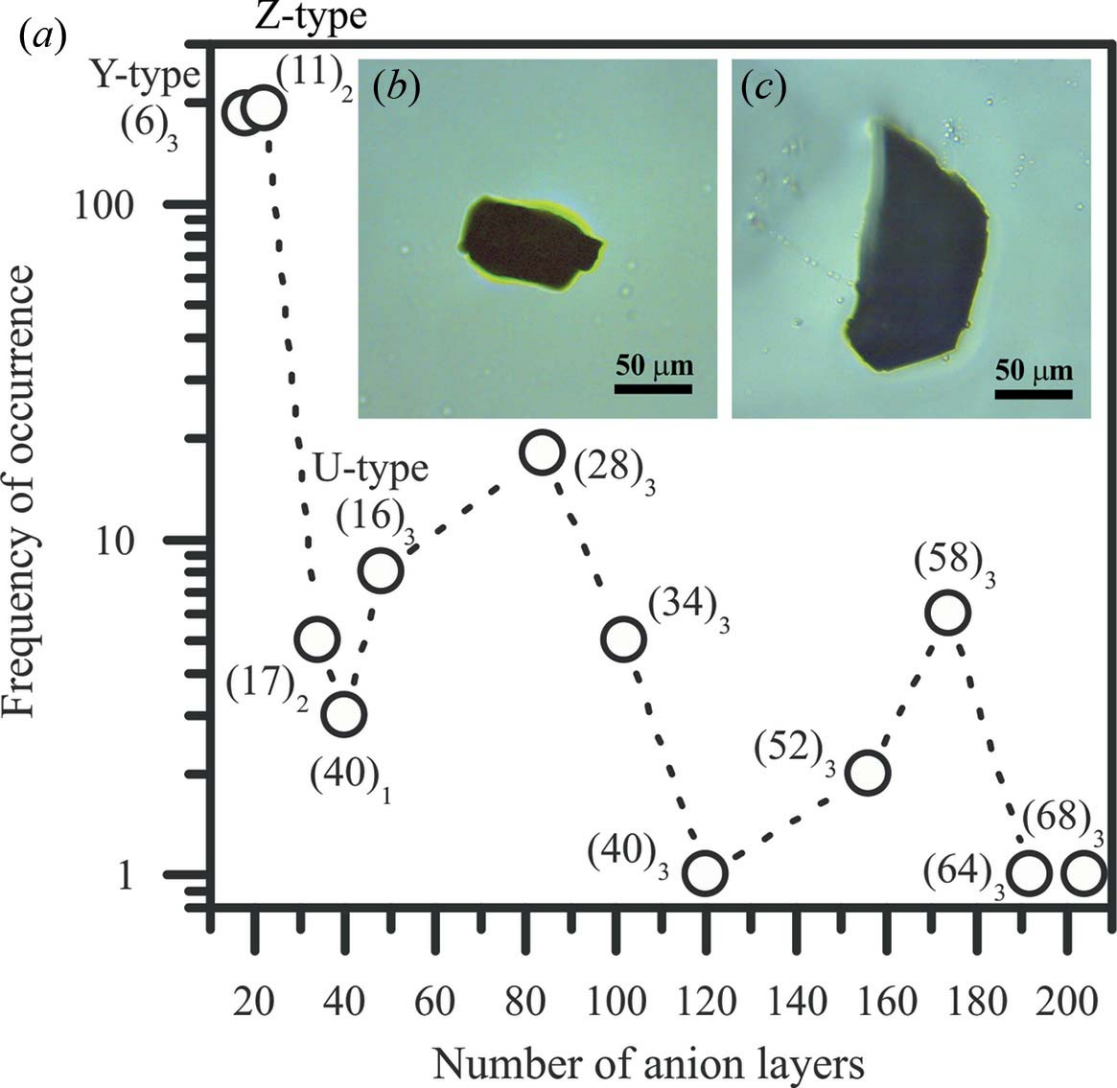
(*a*) Quantity of crystals found as a function of the number of anion layers plotted on a logarithmic scale. The inset shows pictures of mixed-layer hexaferrite crystals belonging to the (*b*) M_2_Y_4_ and (*c*) M_4_Y_8_ series.

This protocol produced black platelet-like single crystals. The resulting batches of crystals contained Z- and Y-type crystals as major phases and some targeted mixed-layer crystals in a minor proportion. Unfortunately, all these different crystal types exhibit the same morphology and aspect (Fig. 3 ▸). The targeted crystals were thus found by a screening process using single-crystal X-ray diffraction to find the *c*-axis value for each single crystal in order to determine the category they belong to. From this screening process, the ratio of Z- and Y-type crystals to mixed-layer hexaferrites crystals was found to be approximately 9:1.

As previously demonstrated by electron and optical microscopy (Savage & Tauber, 1967 ▸), the crystals we formed were often twinned. This feature becomes more frequent and pronounced as the crystal size increases. The mixed-layer crystals are micrometre-sized, with an average around 85 × 65 µm and 20 µm thickness (where thickness direction corresponds to the hexagonal *c* axis).

Different synthesis attempts with varying starting compositions, cooling rates or overall weights were tested in order to improve the mixed-layer yield and the crystal size. The best crystal batch obtained (presenting the highest mixed-layer yield, *i.e.* 11% of the targeted mixed-layer phases) followed the synthesis conditions described above with a BaCO_3_: Fe_2_O_3_:ZnO:B_2_O_3_ ratio of 6:8:1:1 and an overall mass of 1 g.

Attempts to synthesize these compounds as ceramics were also carried out by the standard solid-state route. The ranges of synthesis conditions explored encompassed reaction temperatures from 1100 to 1500°C, dwell times from 3 h to 2 weeks and cooling rates from 0.5°C min^−1^ to quenching to room temperature in air. The resulting samples were always a mixture of M-, Y- and W-type hexaferrites and barium iron oxides, like BaFe_2_O_4_ or Ba_3_Fe_26_O_41_ (see Fig. S1 of the supporting information). Difficulties encountered in obtaining pure polycrystalline phases may suggest that there is a limited range of stability and that M-, Y- and W-type hexaferrites are more stable than the targeted mixed-layer phases.

## 4. Results   

In this study we report nine different classes of mixed-layer hexaferrites that are summarized in Table 4 ▸ with their corresponding features. These nine classes belong to six different series: M_2_Y_3_, M_2_Y_4_/M_4_Y_8_, M_2_Y_5_, M_2_Y_7_, M_2_Y_8_ and M_2_Y_9_. These mainly belong to the extensive M_2_Y_*n*_ subfamily, except for the M_4_Y_8_ series which is twice the M_2_Y_4_ cell length. Some of the nine phases belong to the same series. In that case, they are considered as polytypes: these materials have the same chemical composition but different orderings of the stacking elements. The compounds can be formed with a hexagonal, trigonal or rhombohedral symmetry and exhibit unit-cell lengths from 81.24 to 487.18 Å. We did not observe adjacent M blocks in any of our refined crystal structures.

Note that the different mixed-layer compounds will be named using the number of anion layers they are associated with (see Table 4 ▸), following the (*N*)_*x*_ nomenclature where *N* refers to the number of anion layers of the stacking sequence and *x* refers to the *x*-fold repeat of this stacking sequence within a complete unit cell. The multiplication of the stacking sequence by three indicates a rhombohedral symmetry, with subsequent repeats translated in plane by (2/3, 1/3). For instance, the compound belonging to the M_2_Y_3_ series with the (MYMY_2_)_3_ stacking sequence will hereafter be referred to as the (28)_3_ hexaferrite. This means its unit cell contains a threefold repeat of the stacking sequence which is built from 28 anion layers; the number of anion layers is calculated by adding those of the two M and three Y unit blocks (28 = 2 × 5 + 3 × 6, see Section 2).

### 4.1. Frequency of occurrence   

The frequency of occurrence of crystals found for each mixed-layer compound is plotted in Fig. 3 ▸ against the number of anion layers. The frequency sharply decreases above 22 anion layers, which corresponds to the Z-type hexaferrite. Although one peak is observed for the (28)_3_ hexaferrite, the frequency seems to show a general decrease with the increasing number of anion layers. Our statistics are in agreement with those previously reported from 22 to 100 anion layers (Savage & Tauber, 1967 ▸). Indeed, in the same manner, and as mentioned before in the synthesis (see Section 3), Z-type crystals are commonly found among the targeted mixed-layer hexaferrites (about 200 among ∼500 crystals analysed). Also, as many Y-type (18 anion layers) as Z-type crystals were found experimentally; this feature was not mentioned in the previous study. Note that finding crystals with large repeats turned out to be challenging, *e.g.* only one (68)_3_ crystal was found out of about 500 crystals screened.

### 4.2. Chemical compositions   

Experimental compositions were studied by both single-crystal XRD refinements (discussed in Section 4.3) and energy dispersive X-ray (EDX) spectroscopy. In order to perform EDX analyses, a focused ion beam (FIB) was used for sample preparation: the specimens were cut from single crystals in the out-of-plane direction (*i.e.* lamellae perpendicular to the largest crystal face). This led to a 0.08 µm-thick slice with a surface of 10 × 10 µm (where one of the 10 µm dimensions corresponds to the *c* axis).

The (17)_2_, (34)_3_ and (68)_3_ hexaferrites are polytypes and thus must exhibit the same composition; this was confirmed by the experimental EDX cation ratios (Fig. S2), which are in good agreement with the Ba_10_Fe_72_Zn_8_O_126_ composition determined from the synchrotron refinements.

The slight excess of iron observed in the EDX experiments could come from the holder/microscope. Indeed, a similar shift in composition is also detected in EDX measurements under the same conditions for the Z- and Y-type hexaferrites (Fig. S3), and an iron signal is detected when a measurement is performed using a virgin grid.

Refined compositions of all the mixed-layer hexaferrites (determined from synchrotron refinements where a composition constraint was used to keep an average iron oxidation state of +3) are shown on the Ba–Fe–Zn ternary diagrams in Fig. 2 ▸: these compositions are very close to each other and lie between the Z- and Y-type compositions and particularly on the black line which corresponds to the mixed-layer M_*p*_Y_*n*_ compositional line. Mixed-layer compositions correspond to a series of closely separated intermediates between the Z- and Y-type compositions; they can be described as derivatives of the (MY)_2_ sequence Ba_3_Zn_2_Fe_24_O_41_ Z-type hexaferrite by addition of Ba_2_Zn_2_Fe_12_O_22_ Y blocks. This is consistent with the fact that synthesized crystal batches consist of a mixture of Y-type, Z-type and the targeted mixed-layer crystals.

We also note that the flux growth starting composition (represented by the blue square in Fig. S2 and corresponding to Ba_24_Fe_64_Zn_4_O_124_) forming the mixed-layer compounds does not coincide with their actual compositions. Flux-growth synthesis attempts using the actual compositions (blue crosses in Fig. 2 ▸) did not produce any single crystals. This indicates the complexity of the stability domains of the mixed-layer hexaferrites.

### 4.3. Structural features   

Structures with periods of hundreds of Angstroms are not common in inorganic materials. For comparison, it is convenient to compare reduced (Santoro & Mighell, 1970 ▸) triclinic or Niggli cells, listed in Table S1 in the supporting information. The longest cell parameter, *c*_Niggli_, for the hexaferrites reported here ranges from 66.80 to 162.43 Å. From a search of Pearson’s Crystal Data (Villars & Cenzual, 2017/2018 ▸), restricted to phases with reported atomic positions, the largest unit cells are found among intermetallic phases. For instance, some Al–Ta–Cu alloys with cluster-based structures exhibit giant cubic unit cells with volumes up to 365 372 Å^3^ (*a* = 71.49 Å in space group 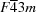, which means the corresponding reduced cell parameter *a*_Niggli_ = 50.55 Å) (Weber *et al.*, 2009 ▸). Samson phases such as NaCd_2_ (*a* = 30.56 Å and *a*_Niggli_ = 21.61 Å; Fredrickson *et al.*, 2007 ▸) and Mg_2_Al_3_ (*a* = 28.49 Å; *a*_Niggli_ = 20.14 Å; Feuerbacher, 2007 ▸) are other examples of the most complicated intermetallic phases known. Hexagonal polytypic materials such as silicon carbide (Verma & Krishna, 1966 ▸), zinc sulfide (Mardix, 1986 ▸) or cadmium iodide (Pałosz, 1983 ▸) also form with large repeats (hexagonal *c*_Niggli_ parameters up to 158.67, 168.71 and 136.70 Å, respectively) and are structurally related to the mixed-layer hexaferrite compounds owing to their hexagonal close packing. Those are also known to generate hundreds of modifications for one composition through different stacking of hexagonal or cubic close-packed layers, although these layers are less chemically diverse than the larger M and Y blocks studied here. In terms of oxide materials, the largest repeat found belongs to a tetragonal structure (*c*_Niggli_ = 69.22 Å) in the Tl–Ba–Ca–Cu–O phase diagram (Hopfinger *et al.*, 2002 ▸). Then comes a series of phases belonging to the Ba–Fe–Ti–O system that are closely related to the mixed-layer hexaferrites discussed here, which form with primitive repeats up to 61.41 Å and combine structural features from barium polytitanates and hexaferrites. Indeed, these compounds are also built from a close packing of {O} and {Ba,O} layers with a transition metal occupying octahedral and tetrahedral interstices (Siegrist *et al.*, 1998 ▸, 2000 ▸, 2002 ▸). Spinel blocks and T blocks are also present in these materials. The former longest fully characterized hexaferrite in the ICSD is the Z-type first reported by Braun (1957 ▸) (*c*_Niggli_ = 52.3 Å).

**Figure 4:**
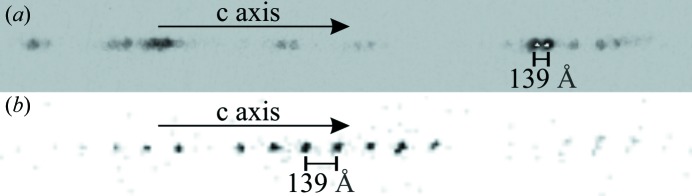
Single-crystal XRD frames of the (58)_3_ hexaferrite collected using (*a*) the Rigaku diffractometer with a maximum detector distance of 137 mm and (*b*) the I19 synchrotron beamline with a 300 mm detector distance. Both frames illustrate the 139 Å primitive repeat of the (58)_3_ hexaferrite.

The structural refinement of such large unit cells was challenging. As a result of the long hexagonal repeats, reflection overlap was prevalent when using the in-house diffractometer (Fig. 4 ▸ *a*). Indeed, data collected to give the highest resolution possible (0.1° scans, short exposure times as the crystals diffract strongly, maximum detector distance of 137 mm, 1 × 1 detector binning mode to give the highest spatial resolution) resulted in structures that are only partially solved, even though data reductions looked sensible (resolution ≤ 0.8 Å, *I*/σ > 20 and *R*_int_ < 0.1). Note that the in-house data were still suitable for performing the screening process. To solve the structures, synchrotron instrumentation was needed, so data were also collected on the I19 beamline at Diamond Light Source (Allan *et al.*, 2017 ▸). This enabled the use of a 300 mm detector distance and the acquisition of well separated reflections (Fig. 4 ▸ *b*). I19 data collections were essential and allowed the determination of these mixed-layer structures. The mixed-layer structural models were then solved and refined with synchrotron X-ray diffraction data. Frames were processed and integrated with the *Xia2* program (Evans, 2006 ▸; Evans & Murshudov, 2013 ▸; Winn *et al.*, 2011 ▸; Winter, 2010 ▸). Data were scaled and corrected for absorption using *SADABS* (Sheldrick, 2008*a* ▸). The space-group determination was performed using *XPREP* software from the *SHELX* package (Sheldrick, 2008*b* ▸). Structures were then solved using *SHELXT* (Sheldrick, 2015 ▸). Structural models were finally refined with *OLEX* ^2^ crystallography software (Dolomanov *et al.*, 2009 ▸).

The first obtained structural model exhibits a chemical composition corresponding to the ideal one without zinc substitution [*e.g.* in the (34)_3_ hexaferrite case, the initial composition input in *SHELX* is Ba_10_Fe_80_O_126_ instead of Ba_10_Zn_8_Fe_72_O_126_]. The refinement procedure was then carried out as follows. All atomic positions were refined, first with isotropic displacement parameters and then with anisotropic displace­ment parameters. At this stage, some iron atoms display unreasonably small anisotropic displacement parameter values, indicating incorrectly assigned atom types. These abnormal values highlight the atomic positions where zinc occupancy is expected; zinc atoms were thus introduced into the structural model at those sites. Attempts to refine these sites with only zinc occupancy led to high anisotropic displace­ment parameter values; a mixed iron/zinc occupancy gave the best results. Note that introducing zinc into the initial composition led to poor structure solutions. The reported final structural models were obtained following this refinement procedure.

### 4.4. The (34)_3_ mixed-layer hexaferrite   

The (34)_3_ mixed-layer hexaferrite shares common structural features with all the studied phases and is moreover a polytype of the new (68)_3_ mixed-layer hexaferrite described later. Therefore, the (34)_3_ hexaferrite, whose magnetic properties were characterized, will be discussed as a typical example.

The structure solution of the (34)_3_ hexaferrite led to a centrosymmetric structure with rhombohedral symmetry (space group 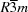) and lattice parameters of *a* = 5.8704 (1) and *c* = 243.5953 (9) Å. This model results in the formula unit Ba_10_Fe_72_Zn_8_O_126_, which corresponds to the ideal structure formula obtained by adding M- and Y-block formulae (two BaFe_12_O_19_ plus four Ba_2_Zn_2_Fe_12_O_22_). Structural refinement gives final agreement factors *R*_1_ = 4.62% and a goodness-of-fit of 1.059. A summary of the structure-refinement results is given in Table 5 ▸.

**Figure 5:**
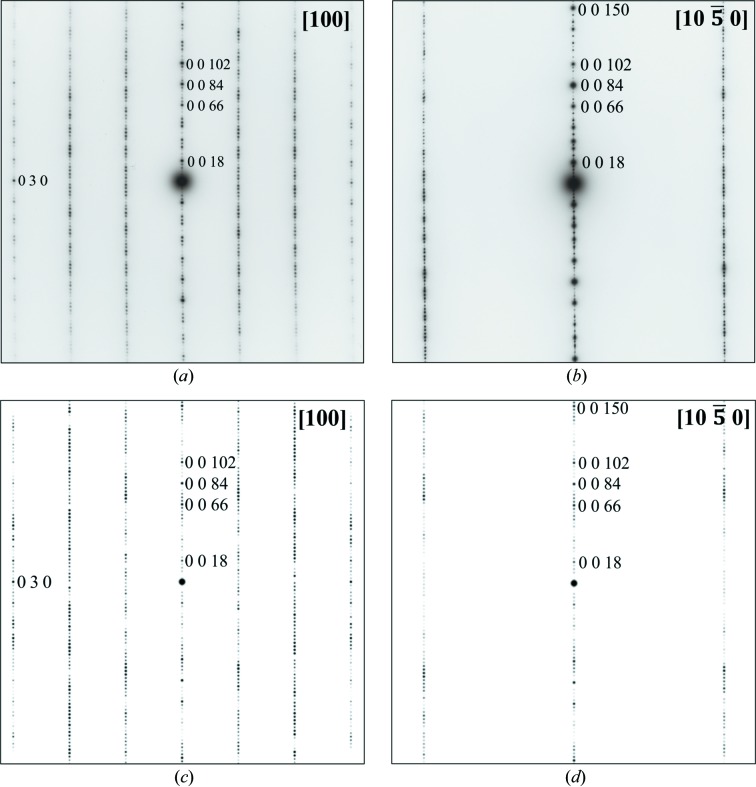
Experimental (*a*) [100] and (*b*) [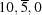] ED patterns and (*c*), (*d*) their corresponding simulated patterns based on the (34)_3_ hexaferrite structural model refined from single-crystal synchrotron X-ray diffraction.

In parallel with the single-crystal XRD investigation and in order to confirm the structural model, the sample was studied by electron diffraction (ED). To perform ED analyses, a focused ion beam (FIB) was used for sample preparation: the specimen was cut from the same single crystal on which synchrotron data were collected, in the out-of-plane direction (*i.e.* lamellae perpendicular to the largest crystal face). The structure solved from synchrotron data is reinforced by the ED study. Indeed experimental ED patterns fit simulations generated from the refined structural model (Fig. 5 ▸): extinction rules are consistent and, as expected, we observe the primitive 81.2 Å repeat along the *c* axis (*l* = 3*n*). Some experimental spot intensities do not perfectly correspond to the simulated ones. This is expected considering multiple scattering and dynamical effects. The indexed most intense simulated spots match the experimental ones.

**Figure 6:**
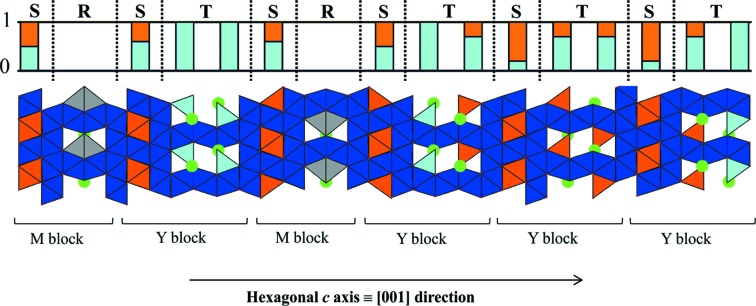
Partial projection of the (34)_3_ hexaferrite structural model corresponding to the MYMY_3_ sequence. The unit cell contains a threefold repeat of the sequence shown, with subsequent repeats translated in plane by (2/3, 1/3). The tetrahedral (light blue), octahedral (dark blue) and bipyramidal (grey) iron environments are represented. Tetrahedral mixed iron/zinc sites are shown in orange and barium atoms are shown in green. The histogram indicates the proportions of iron (light blue) and zinc (orange) occupancy in the tetrahedral sites.

The (34)_3_ hexaferrite can be described as a layered structure along the [001] direction with a succession of the M and Y blocks described in Section 2 (Fig. 6 ▸). The (34)_3_ hexaferrite has the MYMY_3_ sequence and is in agreement with one of the previously observed sequences within the M_2_Y_4_ series (Kohn *et al.*, 1971 ▸). The structure is composed of tetrahedral and octahedral iron sites, bipyramidal iron sites and tetrahedral mixed iron/zinc sites. The histogram shown in Fig. 6 ▸ describes the proportions of iron and zinc occupancy in tetrahedral environments. Three types of T blocks (sub-blocks of Y blocks) are distinguished: those where all tetrahedral sites are occupied by Fe^3+^, those where all tetrahedral sites are mixed Fe^3+^/Zn^2+^ sites and those where half the tetrahedral sites are mixed Fe^3+^/Zn^2+^ sites and the other half are only occupied by Fe^3+^. The zinc distribution is discussed further in Section 4.7.

The local iron and mixed iron/zinc environments are regular in general throughout the structure, as shown by the refined bond distances (Table S9). The most distorted environments correspond to the external octahedra of the T blocks that exhibit two sets of distances [*e.g.* 3 × 1.929 (4) and 3 × 2.260 (4) Å for atom Fe0K in Table S9], compared with 6 × 2.026 (4) Å at the centre of the three face-sharing octahedra (*e.g.* atom Fe0F in Table S9). This distortion is visible in the fragment illustrated in Fig. 1 ▸(*c*), where the Fe atoms of these particular octahedra are displaced equally in opposite directions away from the shared faces of the T block, and is similar to that seen in the hematite structure, which exhibits distances of 3 × 1.946 (1) and 3 × 2.116 (1) Å (Blake *et al.*, 1966 ▸). The hematite-like octahedral distortion of the T blocks was observed in all the hexaferrite structures in this study.

### 4.5. The (68)_3_ mixed-layer hexaferrite: determination of a new stacking sequence   

The crystal growth experiments led to the isolation of a crystal with a new mixed-layer hexaferrite stacking sequence within the M_4_Y_8_ series, *i.e.* the (68)_3_ hexaferrite. As for all other mixed-layer hexaferrites reported here, a combination of synchrotron X-ray diffraction and transmission electron microscopy techniques were used to solve and study the crystal structure of the (68)_3_ hexaferrite.

**Figure 7:**
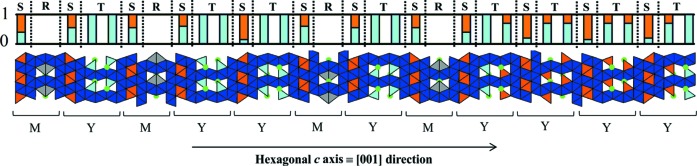
Partial projection of the (68)_3_ hexaferrite structural model corresponding to a single MYMY_2_MYMY_4_ sequence. The unit cell contains a threefold repeat of the sequence shown, with subsequent repeats translated in plane by (2/3, 1/3). The tetrahedral (light blue), octahedral (dark blue) and bipyramidal (grey) iron environments are represented. Tetrahedral mixed iron/zinc sites are shown in orange and barium atoms are shown in green. The histogram indicates the proportions of iron (light blue) and zinc (orange) occupancy in the tetrahedral sites.

The structure solution led to a centrosymmetric structure with rhombohedral symmetry (space group 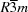) and lattice constants *a* = 5.8721 (1) and *c* = 487.184 (2) Å. The stacking repeat corresponding to this structural model is the new (MYMY_2_MYMY_4_)_3_ sequence. Projections of the stacking sequence and structural parameters are given respectively in Fig. 7 ▸ and Table 6 ▸. Note that the (68)_3_ hexaferrite refined composition, Ba_10_Fe_72_Zn_8_O_126_, corresponds to the (34)_3_ hexaferrite composition, which is expected as these two phases are polytypes.

The structure shows similar features to those described previously for the (34)_3_ hexaferrite. It adopts a layered structure along the [001] direction and is composed of tetrahedral, octahedral and bipyramidal iron sites and of tetrahedral mixed iron/zinc sites. The proportions of iron and zinc occupancy in tetrahedral environments are shown by the histogram in Fig. 7 ▸.

It has been noted that the zinc distribution also differs in the Y blocks, and particularly in its T block part, subject to the layering type (*i.e.* depending on which block types surround the Y block considered). Indeed, the T-block tetrahedral sites are all occupied by zinc atoms in the YYY layers, half or non-occupied in the MYY layers and non-occupied in the MYM layers (Fig. 7 ▸). This Zn^2+^ occupancy trend is similar to that observed for the (34)_3_ hexaferrite. The zinc distribution is discussed in more detail in Section 4.7.

**Figure 8:**
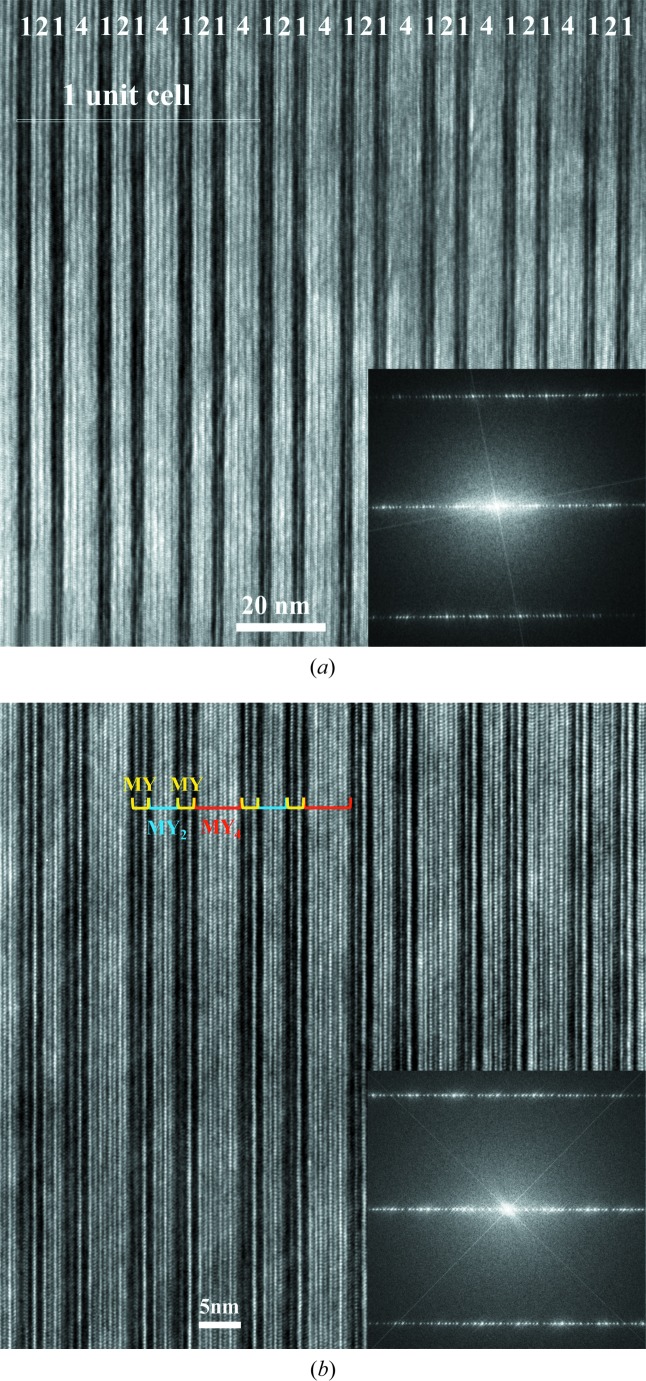
Experimental [100] HREM images collected on an FIB preparation of the (68)_3_ hexaferrite single crystal and its corresponding FFT. These two images highlight the (MYMY_2_MYMY_4_)_3_ stacking sequence. The darker stripes are 25.9 Å long and correspond to ‘MY’ sections. The brighter stripes are related to ‘MY­_2_’ and ‘MY_4_’ sections and are 40.9 and 69.9 Å long, respectively. The (MYMY_2_MYMY_4_)_3_ sequence is indicated through the photograph as ‘1214’ which refers to number succession of Y blocks only. The white rectangle in (*a*) represents one unit cell, containing a threefold repeat of the (MYMY_2_MYMY_4_)_3_ stacking sequence, shown on a shorter length scale in (*b*). The observed periodicity in the FFT is consistent with the primitive repeat of 162 Å.

Kohn *et al.* (1967 ▸) assigned crystals with a 487 Å repeat and with (68)_3_ anion layers to the noncentrosymmetric space group *R*3*m* with the following stacking sequences: (MYMYMY_2_MY_4_)_3_ and (MYMY_2_MY_2_MY_3_)_3_. These sequences were determined by comparing observed and calculated 00*l* intensities. In our case, the new (MYMY_2_MYMY_4_)_3_ stacking sequence, obeying the centrosymmetric space group 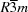, has been determined by quantitative refinement of a full set of synchrotron X-ray diffraction data. This sequence was also shown by high-resolution imaging, as illustrated in Fig. 8 ▸, where the (MYMY_2_MYMY_4_)_3_ repeat is well established and is in agreement with the structural model deduced from synchrotron X-ray diffraction.

### 4.6. Other mixed-layer hexaferrites   

Besides the new (68)_3_ hexaferrite sequence, structural reinvestigation of the eight remaining mixed-layer hexaferrites synthesized confirmed the formation of stacking sequences claimed previously (Kohn & Eckart, 1964 ▸, 1965*a* ▸,*b* ▸, 1967 ▸) (Table 4 ▸). These eight hexaferrites, as exemplified before with the (34)_3_ and (68)_3_ phases, correspond to layered structures built from the same R, S and T structural unit blocks. Each particular assembly of these blocks gives one characteristic compound with a well ordered structure. For all of them, we have precisely defined the crystal structures and determined the location of the Zn^2+^ cations. Note that polytypism is observed within the M_2_Y_4_/M_4_Y_8_ and M_2_Y_5_ series.

**Figure 9:**
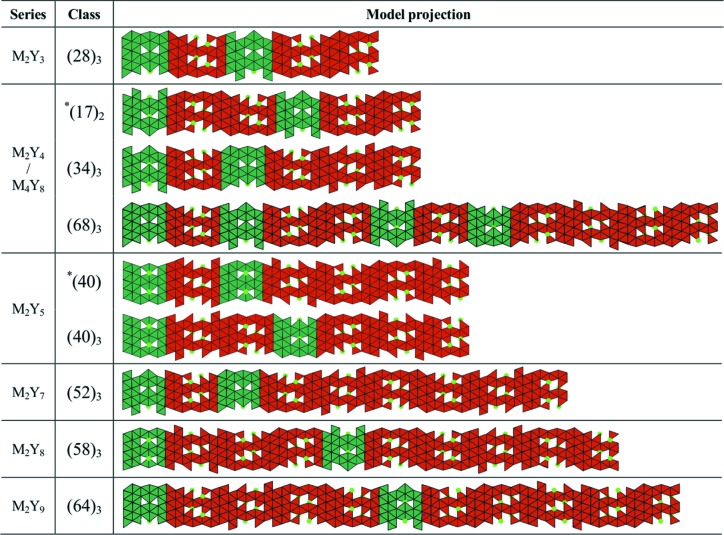
Projections of the mixed-layer hexaferrites along the [100] direction highlighting the different stacking sequences. Green and red blocks correspond to M and Y blocks, respectively. The asterisk indicates a class with hexagonal or trigonal symmetry, all others are rhombohedral. For the latter, unit cells contain a threefold repeat of the represented sequences, with subsequent repeats translated in-plane by (2/3, 1/3).

Details of the refinements for each of the seven mixed-layer hexaferrites that have not been described yet are given in Tables S2–S8. Their corresponding stacking sequences are also illustrated in Fig. 9 ▸. The only observed stacking rule for M and Y blocks is that M blocks may not be adjacent to each other.

### 4.7. Zinc distribution through the mixed-layer structures   

Throughout the entire mixed-layer structures, the Fe^3+^/Zn^2+^ cation order and the zinc occupancy trend have been determined. For all mixed-layer structures, the Fe:Zn ratio was fixed in order to obtain an average iron oxidation state of +3. Note that structures were also considered with a freely refined Fe:Zn ratio. In that case, reliability factors are similar and the final compositions, showing a reduced zinc content of about 30% with respect to the nominal composition, led to an average iron oxidation state of +2.96 (1), suggesting the presence of Fe^2+^ species. For example, in the (34)_3_ hexaferrite, refinements with a fixed Fe:Zn ratio led to the Ba_10_Fe_72_Zn_8_O_126_ final composition, whereas refinements with a Fe:Zn ratio freely refined end up with a final composition of Ba_10_Fe_74.8_Zn_5.3_O_126_. Refinements with a fixed Fe:Zn ratio are chemically reasonable, as hexaferrite compounds are known to be Fe^3+^-based oxides. Moreover, their reliability factors do not significantly differ from those obtained with a freely refined Fe:Zn ratio. This lack of Zn when freely refined could be a result of the complexity of the mixed-layer structures and the fact that iron and zinc have similar atomic scattering factors.

**Figure 10:**
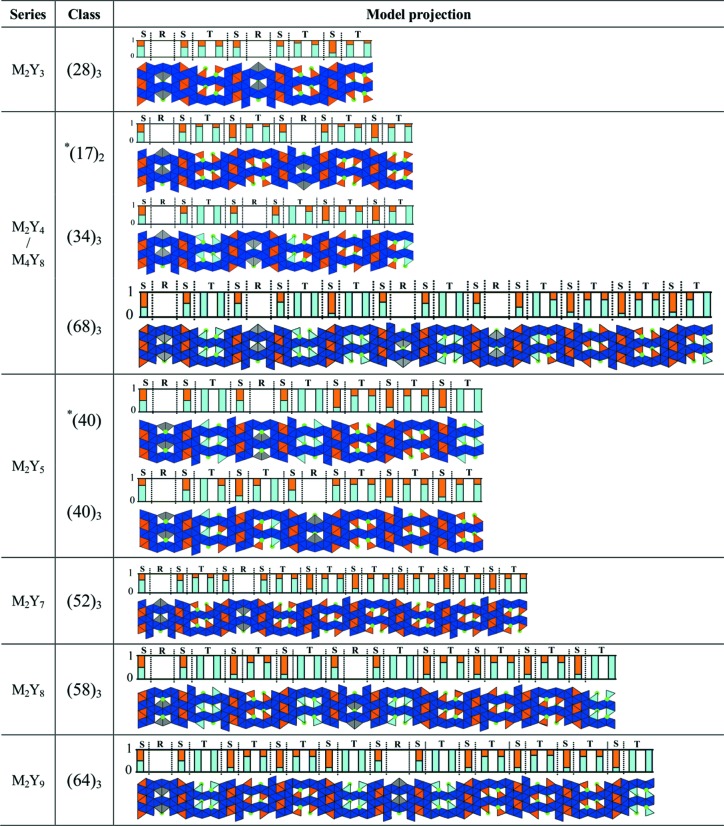
Projections of the mixed-layer hexaferrites along the [100] direction, the location of zinc is highlighted in orange. The asterisk indicates a class with a hexagonal or trigonal symmetry, all others are rhombohedral. For the latter, unit cells contain a threefold repeat of the represented sequences, with subsequent repeats translated in-plane by (2/3, 1/3). The histograms show iron (light blue) and zinc (orange) occupancies in tetrahedral environments.

In terms of location, zinc is found in the mixed iron/zinc tetrahedral sites of the S and T blocks. S-block tetrahedral sites are all occupied with a certain percentage of zinc atoms in all mixed-layer structures, whereas T-block tetrahedral sites are either completely occupied (*i.e.* all sites are mixed Fe^3+^/Zn^2+^ sites), half occupied (*i.e.* half mixed Fe^3+^/Zn^2+^ sites and half Fe^3+^ sites) or non-occupied (*i.e.* all sites are Fe^3+^ sites) depending on the class considered. Note that attempts to force the introduction of zinc onto these unpopulated T-block tetrahedral sites (where only iron was refined) led to either unstable refinements or zinc occupancies smaller than the associated estimated standard deviations. The zinc location throughout the different mixed-layer hexaferrite structures is illustrated in Fig. 10 ▸.

**Figure 11:**
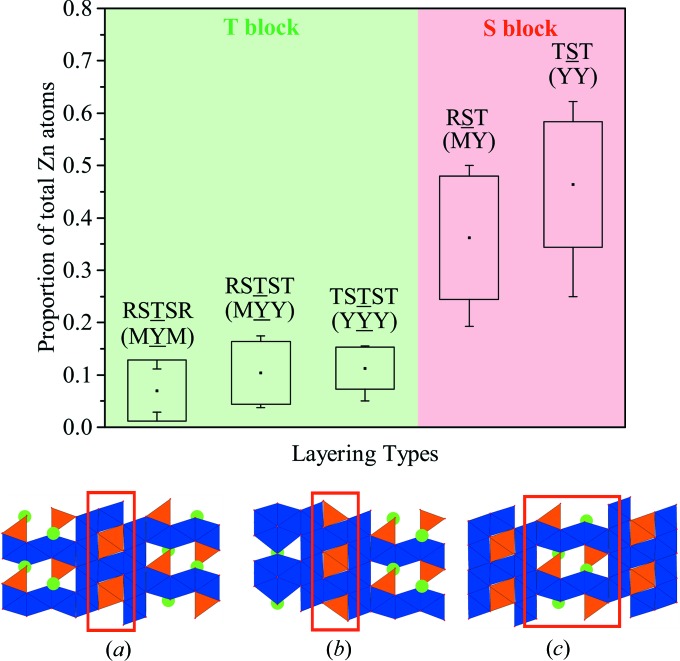
For each hexaferrite, we have determined the number of zinc atoms located in the layer types: RSTSR, RSTST, TSTST, RST and TST. We have plotted the fraction of the total number of zinc atoms in each hexaferrite that is located in that given layer, and averaged over all nine of the mixed-layer structures that we have characterized. The boxes denote one standard deviation, with the whiskers at the minimum and maximum of each range. The (*a*) TST, (*b*) RST and (*c*) STS layering types are shown considering their first neighbours.

In terms of occupancy, these mixed iron/zinc sites are fully occupied, but with different Fe/Zn ratios, as shown by the histograms in Fig. 10 ▸. In order to illustrate the percentage zinc occupancy, we need to consider and differentiate each possible layering type. These are the following: RSTSR, RSTST, TSTST, RST and TST (underlined blocks are those where the zinc occupancy will be evaluated). In terms of M and Y blocks, these five different layers can be described as MYM, MYY, YYY, MY and YY, respectively. For each hexaferrite, we have determined the number of zinc atoms in each of these layers. In turn, this yields the fraction of zinc atoms in a hexaferrite that are located in each layer type. These fractions, averaged over the nine hexaferrites, are plotted in Fig. 11 ▸.

The majority of zinc atoms in each hexaferrite occupy S rather than T blocks – on average, 83±5% of the total number of zinc atoms are located in S blocks, whilst 17±5% are in T blocks. A higher proportion of zinc is expected in the spinel S blocks as they are positively charged if fully occupied by Fe^3+^ (see Section 2). Substitution of Zn^2+^ ions then neutralizes the charge of these blocks. There is also a preference for zinc atoms to occupy TST layers rather than RST layers, with an average of 46±12% of the zinc atoms in TST layers compared with 36±12% in RST layers. The TST layers occur where there are two neighbouring Y blocks (YY ≡ TSTS) and the RST layers occur where an M block neighbours a Y block (MY ≡ RSTS). When splitting out T blocks into the three different layering types and excluding hexaferrites that do not contain those layers (since no zinc atoms can occupy a layer type that is not present in a particular hexaferrite), there is little difference in the fraction of zinc atoms in each layer, although the RSTSR layer type is occupied by a slightly smaller number of zinc atoms. The T blocks are charge neutral when fully occupied by Fe^3+^ (see Section 2), and substitution of Zn^2+^ ions would make them negatively charged.

The only available data about the zinc distribution in the Ba–Fe–Zn–O mixed-layer hexaferrite subgroup is based on Mössbauer studies of the Z-type compound that solely indicates a preferential occupation of Zn ions in the tetrahedral sites (Lim & Kim, 2014 ▸, 2015 ▸; Lim *et al.*, 2017 ▸). Nevertheless, one report on the Y-type hexaferrite deals accurately with the zinc distribution (Collomb *et al.*, 1989 ▸). In the Y-type structure, TST and TSTST are the only two layering types present and this study shows that their tetrahedral sites contain 71±4% and 26±2% of Zn^2+^, respectively. This is consistent with our results (46±12% and 11±4%, respectively), demonstrating that zinc is preferentially located in in the TST blocks.

In order to gain a greater understanding of the location of zinc atoms in our mixed-layer hexaferrites, we studied the zinc distribution computationally. For an individual hexaferrite, combinations of symmetrically related groups of tetrahedral sites were occupied by Zn^2+^, such that the overall crystal symmetry was retained. The number of Zn^2+^ ions introduced was chosen such that charge neutrality was achieved with all Fe ions being in the 3+ charge state, as was the case for the experimental materials. For each combination, we have evaluated the internal energy of the structure using classical force fields. This allows us to determine whether or not the observed preference in Fig. 11 ▸ for zinc atoms to occupy S blocks rather than T blocks is because these structures are more energetically favourable.

**Figure 12:**
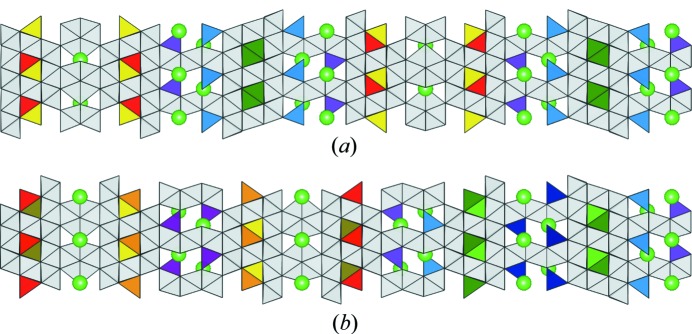
(*a*) Tetrahedral sites for the (17)_2_ hexaferrite. Each colour refers to one of five groups of symmetry-related tetrahedra, with all other polyhedra shown in white. We can see that two groups lie in RST layers (Fe05 shown in red and Fe06 in yellow), two groups lie in T blocks (Fe07 shown in blue and Fe0A in purple) and the Fe (green) group lies in a TST layer. (*b*) Tetrahedral sites for the (34)_3_ hexaferrite. The unit cell contains a threefold repeat of the sequence shown, with subsequent repeats translated in-plane by (2/3, 1/3). Each colour refers to one of ten groups of symmetry-related tetrahedra, with all other polyhedra shown in white. We can see that four groups lie in RST layers (Fe08 shown in red, Fe0B in gold, Fe0I in orange and Fe0S in yellow), four groups lie in T blocks (Fe0E shown in dark blue, Fe0J in light blue, Fe0O in dark purple and Fe0Q in light purple) and two groups lie in TST layers (Fe1A shown in dark green and Fe1 in light green).

We began by identifying each of the tetrahedral sites in the hexaferrite, and then collating them into symmetry-related groups of tetrahedra. Fig. 12 ▸ shows the tetrahedral sites in the (17)_2_ and (34)_3_ hexaferrites, respectively, with each colour referring to a symmetry-related group. These groups are identified by a single site label in the CIFs for the refined structures. For each group, we then identified whether the tetrahedral sites are in T blocks, S blocks surrounded by T blocks (TST layering), or S blocks surrounded by an R block and a T block (RST layering). Having already determined the number of zinc atoms, we next established the number of groups required to accommodate the zinc atoms such that all of the tetrahedral sites in each group are occupied by a zinc atom. From this, we determined the number of combinations of populating zinc atoms amongst the symmetry-related groups of tetrahedral sites for the hexaferrite. We have focused our computational studies on the (17)_2_ and (34)_3_ hexaferrites since there are a manageable number of combinations for these structures: 10 and 210, respectively. We calculate the internal energy by optimizing the atomic positions in each structure to minimize the internal energy, using classical force fields (Gale & Rohl, 2003 ▸). For the (17)_2_ hexaferrite, Fig. 12 ▸(*a*) shows the five symmetry-related groups of tetrahedra, two of which lie in RST layers, two in T blocks and one in a TST layer. Each of these groups contains four tetrahedral sites, and there are eight zinc atoms in the hexaferrite, so that each structure has zinc atoms distributed across two of the five symmetry-related groups. For the (34)_3_ hexaferrite, there are ten symmetry-related groups of tetrahedra, shown in Fig. 12 ▸(*b*). Four of these are in RST layers, four are in T blocks and the remaining two groups are in TST layers. There are six tetrahedral sites in each of these groups, so that each structure features the 24 zinc atoms placed in the tetrahedral sites of four of the ten groups.

**Figure 13:**
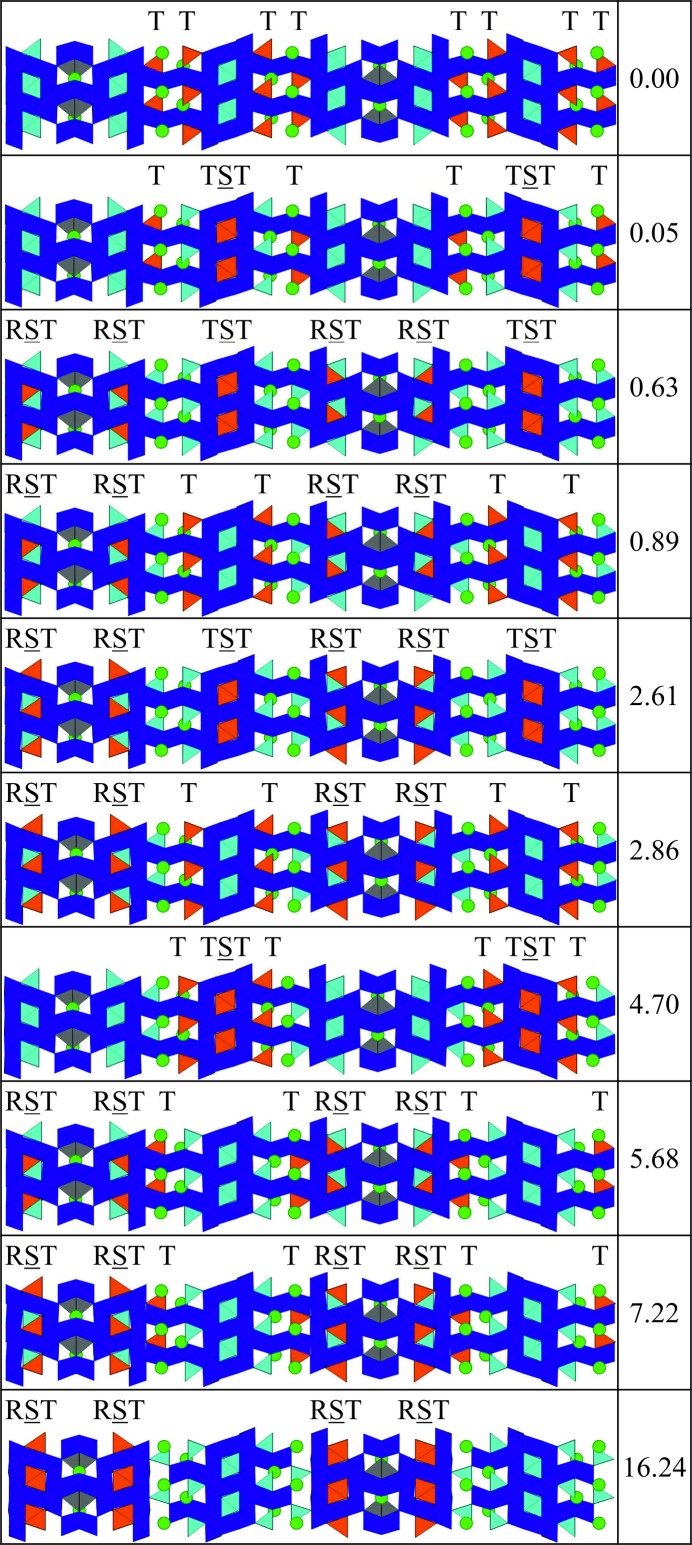
The structures generated by populating Zn atoms in different symmetry-related groups of tetrahedral sites for the (17)_2_ hexaferrite, with their computed energies above the most stable configuration in eV. In each structure, the eight zinc atoms are distributed over two groups of symmetry-related tetrahedral sites, each of which contains four tetrahedra (coloured orange). Fe octahedra are shown in dark blue, with bipyramids in grey and tetrahedra in light blue.

Figs. 13 ▸ and 14 ▸ show the locations of the zinc atoms in the ten lowest-energy structures for the (17)_2_ and (34)_3_ hexaferrites, respectively, along with the relative internal energies of the structures. For the (34)_3_ hexaferrite, a histogram of the energies of all of the structures is shown in Fig. S4. For both hexaferrites, we can see that the lowest-energy structures have zinc atoms in T and TST blocks. In particular, the structure with all of the zinc atoms located in T blocks is low in energy, which is contrary to what is observed experimentally. Conversely, structures with a large proportion of RST layers are high in energy, for example, in the (17)_2_ hexaferrite, the structure with all of the zinc atoms located in RST layers is the least stable and lies 9.02 eV above the next highest-energy structure.

**Figure 15:**
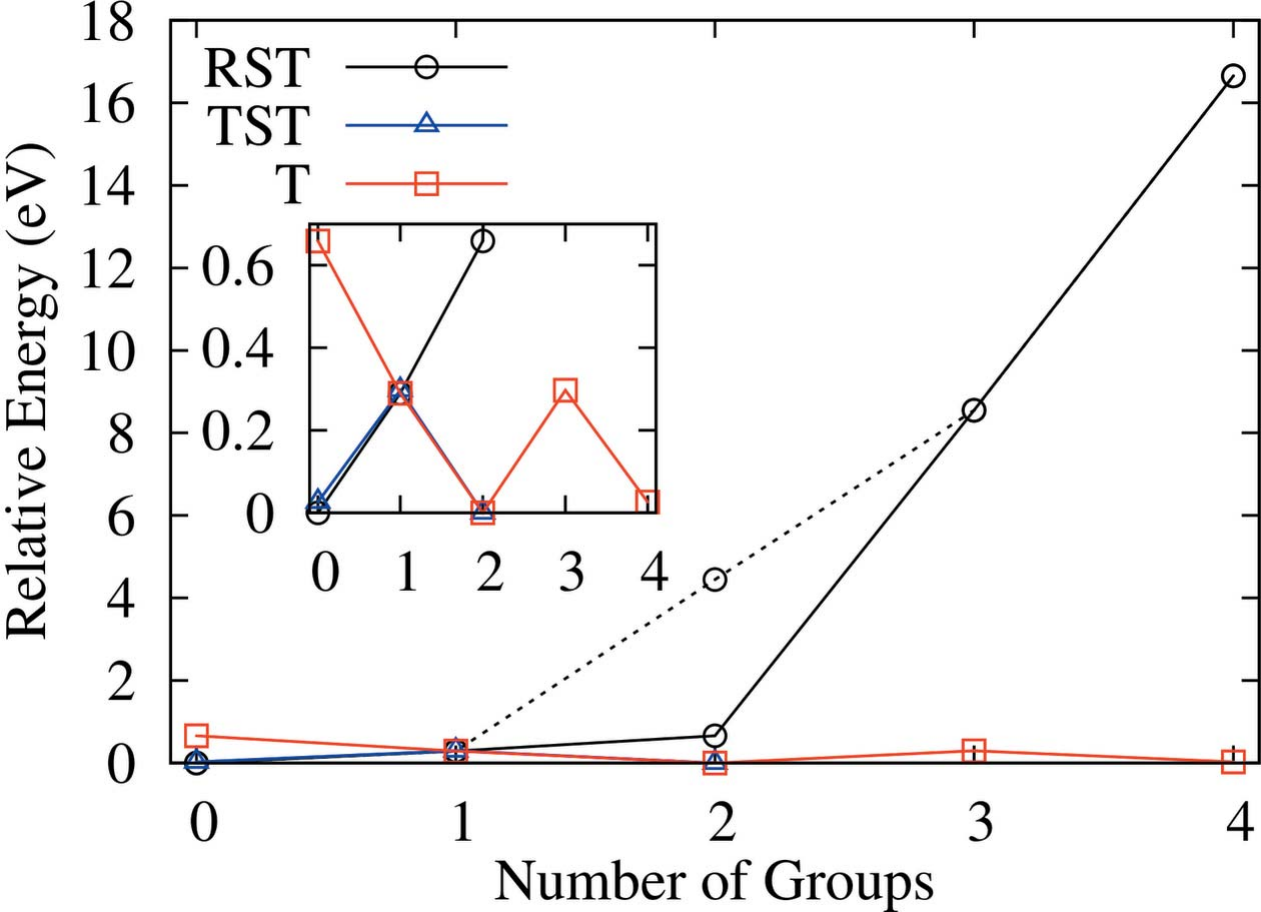
The energy increase compared with the lowest-energy structure for placing zinc atoms in the given number of RST (black), TST (blue) and T (red) groups of symmetry-related tetrahedra for the (34)_3_ hexaferrite. The low-energy values are shown in the inset. Lines are a guide for the eye, with the dashed black line indicating a point representing two RST groups with the additional constraint that the two tetrahedral sites are located within the same RST layer.

These trends are shown in Fig. 15 ▸, which plots the relative energy of the lowest-energy structure with zinc atoms in the given layer types compared with the overall lowest-energy structure for the (34)_3_ hexaferrite. We find that the relative energy for structures with zinc atoms in any number of the available T or TST layers never exceeds 0.7 eV. For RST layers, we observe two different regimes. When placing zinc atoms in one group of RST tetrahedra, the associated energy increase is also less than 0.7 eV. However, there is a significant cost in energy when placing zinc atoms into more than two RST groups, with a relative energy of 8.5 eV for the structure with zinc in three RST groups, and 16.7 eV for four RST groups. Each S block within an RST layer contains two sets of tetrahedra, those closest to the R block and those closest to the T block. When placing zinc atoms into RST blocks, either a single set of these tetrahedra are occupied (*e.g.* red and orange in Fig. 12 ▸ *b*) or both sets of tetrahedra on the same RST layer can be occupied (*e.g.* red and gold in Fig. 12 ▸ *b*). In Fig. 15 ▸, the case where the tetrahedra are on different RST layers is plotted on the solid line, and the case where the two tetrahedral groups are in the same RST layer is plotted on the dashed line. It becomes immediately apparent that it is extremely energetically unfavourable to put the zinc atoms in both sets of tetrahedra that lie within a single RST layer. The relative energy of this is 4.45 eV compared with 0.65 eV for the case of occupying two sets of tetrahedra in different RST layers. For both hexaferrites studied, we find a low-energy regime when zinc atoms do not occupy both of the tetrahedral sites on an RST block, and a high-energy regime when zinc atoms are placed on both sets of tetrahedral sites of an RST block, allowing us to establish the following rule: zinc atoms can occupy either of the two sets of tetrahedral sites in an RST layer, but not both. In Fig. 10 ▸, it can be seen that this rule is always obeyed for all of the hexaferrites that have been synthesized because the zinc occupancy in RST layers never exceeds 50%.

Although the energies of each of our structures has enabled us to establish the rule that zinc atoms cannot occupy both of the sites in an individual RST layer, they do not explain why zinc atoms prefer to occupy tetrahedral sites in S blocks over those in T blocks. Hence, we have sought a more detailed insight into the preferred locations for zinc atoms in our hexaferrites by examining the electrostatic potentials at each of the tetrahedral sites. We can calculate an electrostatic site potential for each atom which is defined by (Gale & Rohl, 2003 ▸)
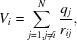
where the potential for atom *i* depends on its neighbours *j*, with *q_j_* the charge on atom *j* and *r_ij_* the distance between atoms *i* and *j*. Hence, this site potential is a measure of the Coulombic interaction per unit charge experienced by an atom at a given position in the structure. We ran further optimizations of the structures of the (17)_2_ and (34)_3_ hexaferrites, where we have first considered structures without any zinc atoms, using iron with an oxidation state of 2.9+ to ensure charge neutrality. By examining the site potentials at each of the tetrahedral sites, we can determine which symmetrically related group of tetrahedra the zinc atoms would preferentially occupy. For the (17)_2_ hexaferrite, zinc atoms occupy the tetrahedral sites in two groups of symmetry-related tetrahedra. Having determined the preferred group, we ran another optimization with Zn^2+^ species in this group, and an iron oxidation state of 2.95+, to determine the group where the remaining zinc atoms would prefer to be located. Similarly, zinc atoms in the (34)_3_ hexaferrite occupy four symmetry-related groups, so we ran a series of optimizations with iron oxidation states of 2.9+, 2.92+, 2.95+ and 2.97+. For each optimization in this series, zinc atoms were placed in the most favourable group identified by the previous optimization. Hence, we approximate the process of accommodating zinc atoms while growing the hexaferrite crystals.

Tables 7 ▸ and 8 ▸ show the average site potential for each of the symmetrically related groups of tetrahedra in the (17)_2_ and (34)_3_ hexaferrites, respectively. When there are no zinc atoms present in the structure, we find for both hexaferrites that TST sites have the least negative site potentials, which indicates that they are the most favourable location for Zn^2+^ species compared with Fe^3+^ species. This observation matches with the experimental structures, given that the zinc occupancies of the TST layers for these hexaferrites are 75 and 80%, respectively (see Fig. 10 ▸). When zinc atoms are introduced onto the TST sites in both hexaferrites (final column of Table 7 ▸, and third, fourth and fifth columns in Table 8 ▸), we immediately note that the ranking of the site potentials changes completely, so the most favourable sites for all of the zinc atoms cannot be ascertained from the calculation without zinc atoms alone. In these columns, we can also see that the potential of the TST site occupied by Zn^2+^ is much less negative compared with when it contained a Fe^2.9+^ species. Of the remaining tetrahedral sites for the (17)_2_ hexaferrite, we find that the site with the least negative potential, and therefore the most favourable location for the remaining Zn atoms, is the RST site where the tetrahedra lie closer to the T block (see Fig. 12 ▸ *a*). For the (34)_3_ hexaferrite, our series of optimizations tells us that the most favourable locations for the zinc atoms are the two sets of TST sites, one of the T blocks, and an RST site where the tetrahedra lie closer to the T block (see Fig. 12 ▸ *b*). Figs. 13 ▸ and 14 ▸ show that the structures with the zinc atoms located on these sites are the third lowest in energy for both hexaferrites, 0.63 eV above the most stable for the (17)_2_ hexaferrite, and 0.29 eV above the most stable for the (34)_3_ hexaferrite. By studying the site potentials, we have seen that the zinc atoms prefer to occupy the tetrahedra in S blocks, in line with the experimental observations. In addition, the structures corresponding to these populations of the zinc atoms best correspond to the structures that are found experimentally.

Our calculations have enabled us to both establish a rule regarding the distribution of zinc atoms in mixed-layer hexaferrites and gain insight into the experimental observations in Fig. 11 ▸. When considering the energy of different combinations of populating zinc atoms onto the groups of symmetrically related tetrahedra for the (17)_2_ and (34)_3_ hexaferrites, we can establish the rule that zinc atoms cannot be placed on both sites in an RST layer, and we find this to be the case in the experimentally observed structures. Turning our attention to the site potentials of tetrahedral sites, we made the important observation that the most favourable sites for zinc atoms changes as we introduce more zinc into the structure and, hence, the most favourable structure is different when populating zinc atoms sequentially compared with populating them simultaneously. During the growth of the hexaferrites, zinc atoms can be locked into locally preferred sites, which may explain why the structures observed do not match the global minimum energy structure. Indeed, we found for the (17)_2_ and (34)_3_ hexaferrites that, when considering the electrostatic site potentials, the most favourable tetrahedral groups for zinc atoms gave the structures that best correspond to the experimentally observed structures, and are also amongst the lowest in energy.

### 4.8. Defects in stacking sequences   

**Figure 16:**
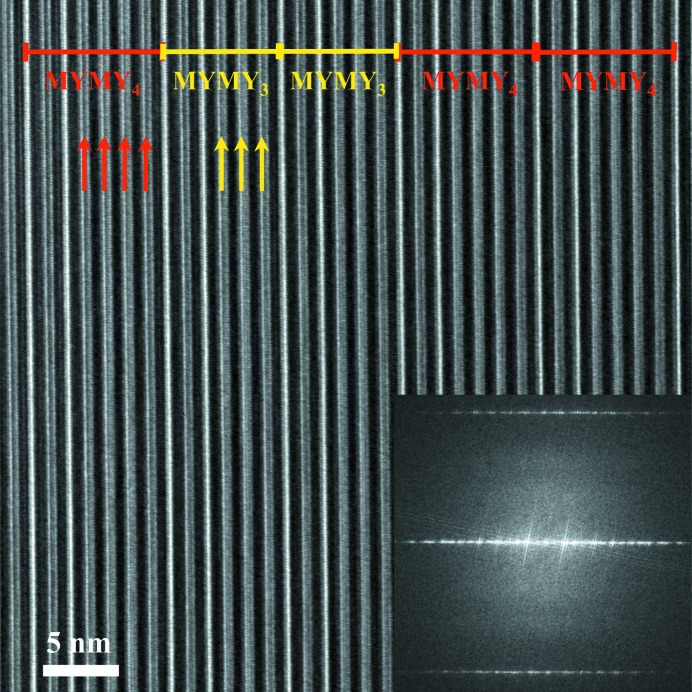
Experimental [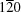] HREM image collected on an FIB preparation of the (40)_1_ hexaferrite single crystal and its corresponding FFT. ‘MYMY_3_’ defects are observed along the stacking sequence. Here, the FFT is not of sufficient resolution to distinguish the global (95 Å) repeat and the defect (81 Å).

Disorder phenomena have been observed within some of the mixed-layer structures and more specifically stacking defects have been detected by high-resolution electron microscopy (HREM) imaging. These defects consist of local areas where deviations from the perfect stacking sequence are observed. For instance, Fig. 16 ▸ shows the (40)_1_ hexaferrite stacking sequence containing isolated faults. For this mixed-layer compound, the MYMY_4_ sequence is expected. On the HREM image, the combination of one white stripe and one dark stripe corresponds to one M block, whereas the combination of one grey stripe and one dark stripe corresponds to one Y block. Red segments then represent the regular (40)_1_ hexaferrite stacking sequence, whereas yellow segments highlight stacking defects. The latter are 163 Å long and formed from the MYMY_3_ sequence. This correlates to two thirds of the (34)_3_ hexaferrite structure repeat.

These stacking defects result in the presence of residual electron-density peaks on structural models in the cases of the (40)_1_, (40)_3_, (34)_3_ and (68)_3_ hexaferrites.

Faults along the stacking sequence are expected in these layered structures considering the growth mechanism and the lengths of the repeats (Cook & Nye, 1967 ▸; Turner *et al.*, 1996 ▸) and have been observed previously in hexaferrite compounds (Van Landuyt *et al.*, 1974 ▸; McConnell *et al.*, 1974 ▸; Anderson & Hutchison, 1975 ▸). These earlier studies highlighted three categories of defects: the incorporation/lack of one or several Y unit blocks, the lack of larger mixed blocks (*e.g.* an ‘MY_4_’ block) and the swapping of blocks (*e.g.* MY_2_MY_3_MY_4_MY_5_ becomes MY_2_MY_4_MY_3_MY_5_). From our observations, the incorporation/lack of one or several Y-unit blocks is the most common defect. A missing mixed-block defect has also been observed but none corresponding to the swapping of blocks.

### 4.9. Magnetic properties of the (34)_3_ hexaferrite   

**Figure 17:**
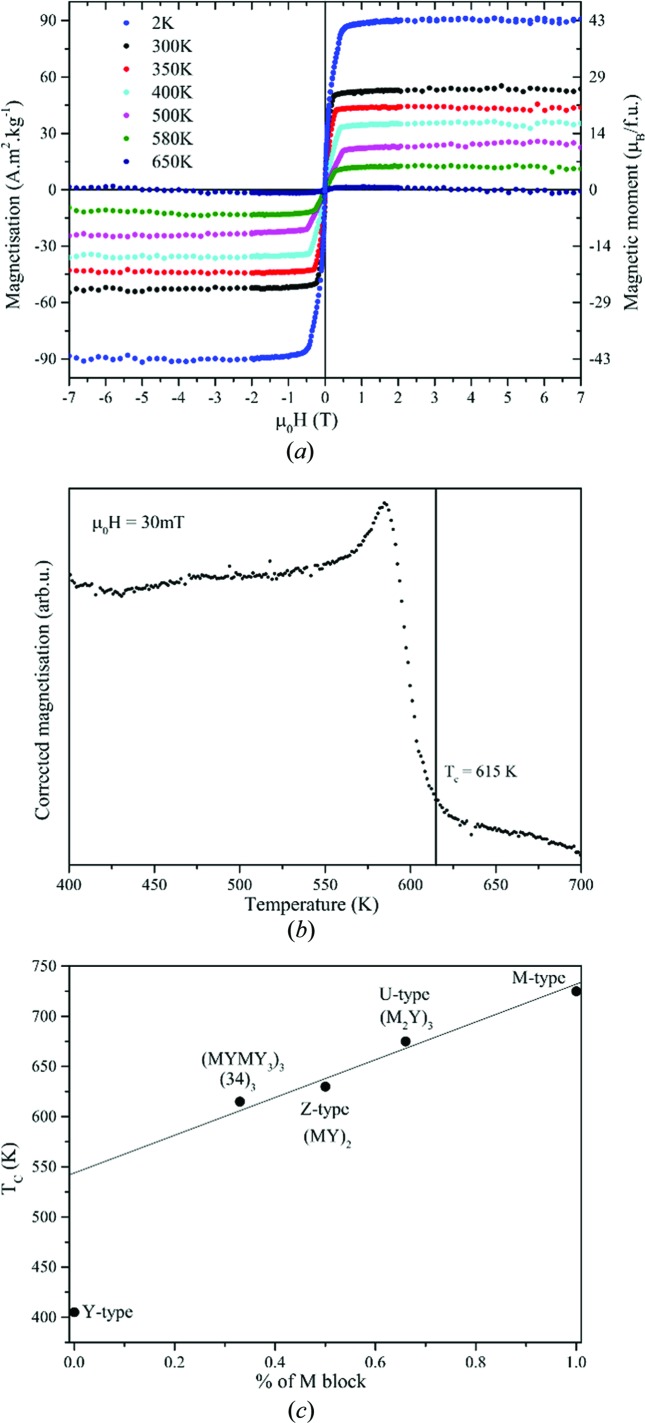
(*a*) Isothermal magnetization as a function of applied magnetic field of the (34)_3_ hexaferrite at selected temperatures. (*b*) Magnetization as a functions of temperature between 400 and 700 K under an external magnetic field of 30 mT. The Curie temperature is highlighted with a vertical line at *T*_C_ = 615 K. (*c*) Curie temperatures of the M-type, Y-type and mixed-layer hexaferrites as a function of the percentage of M block present within their stacking sequences.

Owing to the small crystal size and the possible presence of twins and inclusion of other sequences, all magnetic measurements have been performed twice on two different (34)_3_ mixed-layer hexaferrite single crystals and were reproducible in the value of the Curie temperature and saturation magnetization within the error [which is dominated by the estimation of the crystal mass from the optically measured volume and density (ρ_calc_) calculated from X-ray diffraction]. The isothermal magnetization loops recorded at various temperatures between 2 and 650 K are presented in Fig. 17 ▸(*a*) and clearly indicate some long-range magnetic ordering dominated by ferromagnetic interactions, with an elevated Curie temperature between 580 and 650 K. A saturation magnetization *M*_s_ = 90 (10) A m^2^ kg^−1^ is measured at 2 K, corresponding to a magnetic moment of 1.6 μ_B_ per Fe, which is significantly lower than the value of 5 μ_B_ expected for fully ferromagnetically ordered Fe^3+^, clearly indicating a ferrimagnetic ground state similar to other hexaferrites (Pullar, 2012 ▸). The magnetic moment of the hexaferrite sequence can be calculated using the rule that R and S blocks contribute a net moment of 2 μ_B_, and T blocks contribute 0 μ_B_ (Pullar, 2012 ▸). Using the sequence refined for the (34)_3_ structure presented in this study (Fig. 7 ▸), the theoretical magnetic moment should be 48 μ_B_/f.u., which is close to the value of 43 (4) μ_B_/f.u measured. Measurements were performed with the magnetic field applied perpendicular to the thinnest dimension of the single crystal, *i.e.* perpendicular to the *c*-axis direction. The slight change of slope in the magnetization below the saturation magnetization, together with a large magnetic anisotropy field *H*_A_ = 398 kA m^−1^ (defined as the field required to saturate the magnetization), suggests that the magnetic field is applied along the hard axis of magnetization. This is in agreement with the observation that most hexagonal ferrites have a preferred axis of magnetization along the *c* axis, except for Y ferrites and Co_2_-ferrites, which present a preferred magnetization orientation in the hexagonal basal plane (Pullar, 2012 ▸). The uniaxial anisotropy constant can be estimated using the expression *K*_u_ ≃ (μ_0_ *H*_A_ *M*_s_ρ_calc_)/2 and is found to be 1.2 × 10^5^ J m^−3^, which is similar to the reported values for common hexagonal hexaferrites (Pullar, 2012 ▸). Despite the large uniaxial anisotropy constant, a low coercivity of *H*_C_ = 25 Oe is measured at 2 K and increases with temperature to a value of 300 Oe at 300 K. This is commonly observed in known Z- and U-type mixed-layer hexaferrites (Lim *et al.*, 2017 ▸; Lisjak & Drofenik, 2004 ▸). The low coercivity of the Y-type (*H*_C_ < 100 Oe; Obulesu *et al.*, 2017 ▸; Odeh *et al.*, 2016 ▸) unit block seems to dominate the high coercivity of the M-type (*H*_C_ > 2500 Oe; Pullar, 2012 ▸) unit block. Note that no precise *H*_C_ values are given for M-, Y-, Z- and U-type hexaferrites because these vary considerably with the synthesis procedure in terms of grain size and, in general, a low coercive field is observed in large grains. Magnetic-susceptibility measurements as a function of temperature were carried out from 2 to 700 K under 30 mT. The corrected magnetization as a function of temperature in the range 400–700 K is presented in Fig. 17 ▸(*b*), and the sharp transition observed at 615 K is assigned to the Curie temperature of the compound.

The (34)_3_ hexaferrite sits between the M- and Y-type Curie temperatures: M-type = 725 K and Y-type = 403 K (Pullar, 2012 ▸). This is consistent as the Y content is higher than the M content (M_2_Y_4_ series). The more the Y content increases, the more the resulting *T*_C_ should decrease. This trend is indeed observed when plotting the Curie temperature as a function of the percentage of M block in mixed-layer hexaferrites (Fig. 17 ▸ *c*).

## 5. Conclusions   

Several mixed-layer hexaferrites were isolated as single crystals using a flux-growth method. Their structural solution, carried out by combining synchrotron and TEM analyses, was particularly challenging owing to their large and complex crystal structures (repeats up to 487 Å) and also the rarity of the crystals. Structural reinvestigations revealed the existence of a new stacking sequence within the M_4_Y_8_ series and particularly in the (68)_3_ hexaferrite. Its structural model, established here for the first time, exhibits the largest repeat among hexaferrites, and potentially all metal oxide materials. These structural reinvestigations also allowed the quantitative refinement of a number of previously proposed stacking sequences for eight different compounds and the precise definition of their crystal structures. In addition, the trend of preferred Zn occupancies has been established within the entire MY series presented in this paper. It has been shown that Zn mostly occupies the TST spinel blocks of the mixed-layer structures and that their occupancy is influenced by the nature of the neighbouring block types. This is driven by the most favourable sites for initial occupation by Zn^2+^ according to electrostatic potential, rather than the overall internal energy of a given Zn^2+^/Fe^3+^ configuration. We also established the rule that zinc atoms cannot be placed on both sites in an RST layer. The ferromagnetic *T*_C_ of the (34)_3_ hexaferrite has been determined.

## 6. Experimental   

### 6.1. Optical microscopy   

Single-crystal image captures were performed with a Brunel SP350 microscope.

### 6.2. X-ray single-crystal data collection and analysis   

Details of the single-crystal growth conditions are described in Section 3. Single-crystal X-ray diffraction data were collected at 100 K on a Rigaku MicroMax-007 HF with a molybdenum rotating-anode microfocus source and a Saturn 724+ detector using *CrystalClear* (Rigaku, 2009 ▸).

Synchrotron data were collected at 100 K on the I19 beamline at Diamond Light Source (λ = 0.6889 Å). Single crystals were mounted under inert oil on MiTeGen tips. Crystal-structure resolutions and refinements have been carried out on synchrotron data. *DIAMOND* (Brandenbry, 2006 ▸) was used for graphical representation of the structures. Further details of the data collection and structure solution are described in Section 4.3.

### 6.3. Powder X-ray diffraction   

Routine PXRD characterization was carried out using a PANalytical X’Pert Pro diffractometer in Bragg–Brentano geometry with a monochromated Co *K*α_1_ source (λ = 1.78896 Å) and position-sensitive X’Celerator detector. Details of the attempted powder syntheses conditions are described in Section 3.

### 6.4. TEM-EDX and selected-area electron diffraction   

The SAED patterns were recorded using a JEOL 2000FXII microscope operating at 200 kV. Samples were prepared from raw single crystals using an FEI Helios 600i FIB (Ga) instrument. Thin lamellae were sectioned and mounted on Cu grids using the lift-out technique.

EDX spectroscopy data were also collected using a 200 kV JEOL 2000FXII microscope. EDX spectra were collected at ten different areas on each crystal for several minutes in order to obtain a suitable signal-to-noise ratio. The quantification data for each element were corrected using a correction factor determined from a standard.

### 6.5. High-resolution electron microscopy   

HREM images were recorded using a Schottky field-emission-gun-equipped JEOL JEM 2100FCs microscope operating at 200 kV. Simulated ED patterns were generated using the ‘SingleCrystal’ interface of the *CrystalMaker* software (Rigaku, 2009 ▸).

### 6.6. Magnetic measurements   

Magnetic measurements were carried out on single-crystal samples using a commercial magnetometer MPMS3 (Quantum Design, USA). The measurements from 2–300 K were collected using the VSM mode with the sample glued to a quartz plate with low-susceptibility epoxy and from 300–700 K using the SQUID detection DC mode with the sample glued to the heating sample holder with high-temperature cement. Magnetization as a function of temperature was recorded from 2–700 K in the following modes: ZFC (zero field cooling, measured while warming after cooling in a zero field) and FC (field cooling, measured while warming after cooling under a magnetic field) under a magnetic field of 30 mT. Isothermal magnetization as a function of applied magnetic field was also measured at 2, 300, 400, 500 and 650 K from −7 and 7 T. The data collected from 2–300 K were corrected by subtracting a diamagnetic background from the glue and the data collected from 300–700 K with a combination of diamagnetic and paramagnetic background from the high-temperature cement.

### 6.7. Computation   

Force-field calculations were performed using *GULP* (Gale & Rohl, 2003 ▸). Buckingham short-range potentials were used between cations and anions, and between two oxide ions, with a radial cut-off of 12 Å. The long-range electrostatic energy was calculated with atomic charges split between harmonically coupled cores and shells to model polarization. Unit-cell parameters and atomic positions were optimized until the norm of the gradient was lower than 0.05. All force-field parameters were obtained from the literature (Woodley *et al.*, 1999 ▸; Maglia *et al.*, 2006 ▸). In particular, we investigated two different sets of Zn parameters from the work by Lewis & Catlow (1985 ▸). The parameters we studied are given in Table 9 ▸.

We repeated our optimization of the ten structures shown in Fig. 13 ▸ using both sets of parameters in order to compare the relative energies of the structures. The energies are shown in Table S10. For the first set of parameters, the energy ranking of the structures is identical to that obtained from our original force field, and the relative energies are in good agreement. For the second set of parameters, the rankings of two pairs of structures have swapped (the third and fourth and the fifth and sixth), but the relative energies remain in good agreement and the lowest and highest energy structures are the same.

## 7. Related literature   

The following reference is cited in the supporting information: Togo (2009 ▸).

## Supplementary Material

Crystal structure: contains datablock(s) I, 81a_a, 200a_a, 244a_a, 95a_a, 287a_a, 374a_a, 417a_a, 462a_a, 488a_a. DOI: 10.1107/S2052252518011351/lt5011sup1.cif

Additional figures and tables. DOI: 10.1107/S2052252518011351/lt5011sup2.pdf

CCDC references: 1861101, 1862982, 1862983, 1862984, 1862985, 1862986, 1862987, 1862988, 1862989, 1862990

## Figures and Tables

**Figure 14:**
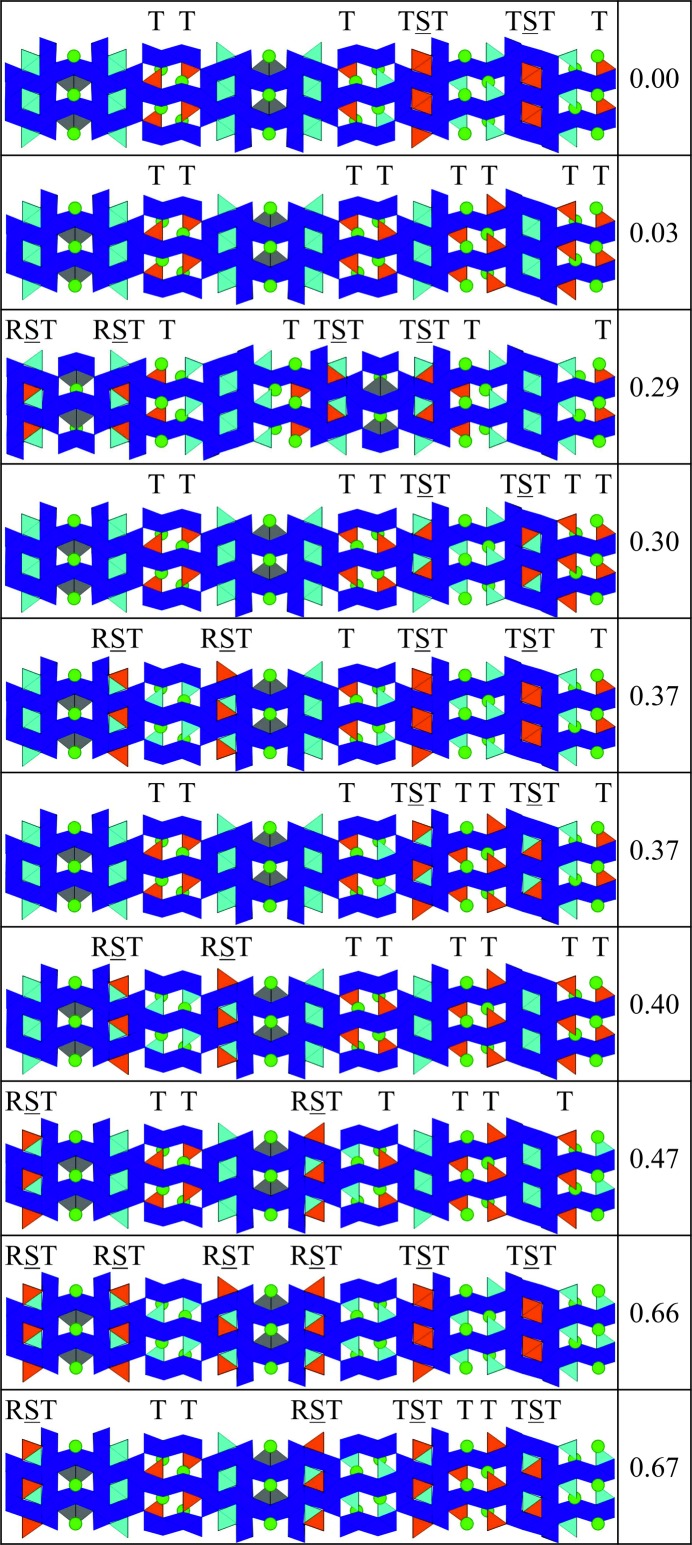
The structures generated by populating Zn atoms in different symmetry-related groups of tetrahedral sites for the (34)_3_ hexaferrite, with their computed energies above the most stable configuration in eV. The unit cells contains a threefold repeat of the sequences shown. In each structure, the 24 zinc atoms are distributed over four groups of symmetry-related tetrahedral sites, each of which contains six tetrahedra (coloured orange). Fe octahedra are shown in dark blue, with bipyramids in grey and tetrahedra in light blue.

**Table 1:** Structural and magnetic characteristics of the M-type and Zn-containing hexaferrites (*e.g.* Zn_2_W = BaZn_2_Fe_16_O_27_) (Pullar, 2012 ▸)

Hexaferrite	Formula	*c* (Å)	Space group	Magnetization at room temperature	*T*_C_ (K)
M	BaFe_12_O_19_	23.18	*P*6_3_/*mmc*	Uniaxial	723
Zn_2_W	BaZn_2_Fe_16_O_27_	32.84	*P*6_3_/*mmc*	Uniaxial	703
Zn_2_Y	Ba_2_Zn_2_Fe_12_O_22_	43.56	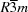	In plane	403
Zn_2_Z	Ba_3_Zn_2_Fe_24_O_41_	52.30	*P*6_3_/*mmc*	Uniaxial	633
Zn_2_X	Ba_2_Zn_2_Fe_28_O_46_	84.11	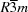	Uniaxial	704
Zn_2_U	Ba_4_Zn_2_Fe_36_O_60_	114.48	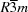	Uniaxial	673

**Table 2:** Description of known hexaferrite unit cells from the R, S and T fundamental unit blocks, and the larger M and Y building blocks We use the (…)_*x*_ nomenclature, where *x* refers to the number of repeated sequences within one complete unit cell.

Hexaferrite type	Structural unit blocks
M	(RS)_2_ = M_2_
Y	(TS)_3_ = Y_3_
W	(RS S)_2_ = (MS)_2_
X	(RS RS S)_3_ = (M_2_M)_3_
Z	(RS TS)_2_ = (MY)_2_
U	(RS RS TS)_3_ = (M_2_Y)_3_

**Table 3:** Partial list of observed M_*p*_Y_*n*_ mixed-layer hexaferrites (Kohn & Eckart, 1965*a* ▸, 1967 ▸; Kohn *et al.*, 1971 ▸) with compositions matching those reported in this study The stacking sequence of M and Y blocks was derived from etched step edges measured with electron microscopy, or the intensities of the 00*l* reflections in one-dimensional X-ray diffraction patterns.

M:Y	Composition (nominal)	Stacking sequences	Space group	*c* axis (Å)
2:4	Ba_10_M_8_Fe_72_O_126_	(MYMY_3_)_3_	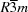	244.0
		(MY_2_)_2_	*P*6_3_/*mmc*	81.3
2:5	Ba_12_M_10_Fe_84_O_148_	(MY_2_MY_3_)_3_	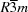	287.6
2:7	Ba_16_M_14_Fe_108_O_192_	(M_2_Y_7_)_3_ and (MYMY_6_)_3_ and (MY_3_MY_4_)_3_	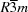	374.7
		(MY_2_MY_5_)_1_	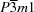	124.9
2:8	Ba_18_M_16_Fe_120_O_214_	(MY_2_MY_6_)_3_ and (MY_3_MY_5_)_3_	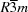	418.5
		(MYMY_7_)_1_	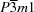	139.4
		(MY_4_)_2_	*P*6_3_/*mmc*	139.4
4:8	Ba_10_M_8_Fe_72_O_126_	(MYMYMY_2_MY_4_)_3_ and (MYMY_2_MY_2_MY_3_)_3_	*R*3*m*	487.8
		(MYMYMY_3_MY_3_)_1_	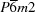	162.6
4:10	Ba_12_M_10_Fe_84_O_148_	(MMYMY_2_MY_7_)_1_	*P*3*m*1	191.6
		(MYMY_2_MY_2_MY_5_)_3_	*R*3*m*	574.9

**Table 4:** Summary of the synthesized mixed-layer hexaferrites

Series	*c* axis (Å)	Space group	Stacking sequence	Number of anion layers	Composition
M_2_Y_3_	200.135 (3)	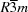	(MYMY_2_)_3_	(28)_3_ = 84	Ba_8_Fe_60_Zn_6_O_104_
M_2_Y_4_/M_4_Y_8_	81.2388 (8)	*P*6_3_/*mmc*	(MY_2_)_2_	(17)_2_	Ba_10_Fe_72_Zn_8_O_126_
	243.5953 (9)	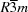	(MYMY_3_)_3_	(34)_3_ = 102	
	487.184 (2)	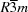	(MYMY_2_MYMY_4_)_3_	(68)_3_ = 204	
M_2_Y_5_	95.725 (1)	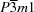	(MYMY_4_)_1_	(40)_1_	Ba_6_Fe_42_Zn_5_O_74_
	287.187 (7)	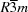	(MY_2_MY_3_)_3_	(40)_3_ = 120	
M_2_Y_7_	374.176 (1)	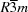	(MYMY_6_)_3_	(52)_3_ = 156	Ba_8_Fe_54_Zn_7_O_96_
M_2_Y_8_	417.644 (1)	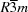	(MY_3_MY_5_)_3_	(58)_3_ = 174	Ba_9_Fe_60_Zn_8_O_107_
M_2_Y_9_	461.224 (1)	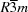	(MY_4_MY_5_)_3_	(64)_3_ = 192	Ba_10_Fe_66_Zn_9_O_118_

**Table 5:** Details of the single-crystal structure refinement for the (34)_3_ hexaferrite

Series	M_2_Y_4_
Number of anion layers	(34)_3_ = 102
Formula	Ba_10_Fe_72_Zn_8_O_126_
Crystal system	Trigonal
Space group	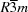
*a* = *b* (Å)	5.8704 (1)
*c* (Å)	243.5953 (9)
*V* (Å^3^)	7270.01 (3)
*Z*	3
Sequence	(MYMY_3_)_3_
ρ_calc_ (Mg m^−3^)	5.404
*T* (K)	100
μ (mm^−1^)	14.127
Shape, colour	Platelet, black
Size (µm^3^)	58 × 58 × 14
λ (Å)	0.6889
*R*_1_	0.0462
Goodness-of-fit	1.059

**Table 6:** Details of the single-crystal structure refinement for the (68)_3_ hexaferrite

Series	M_4_Y_8_
Number of anion layers	(68)_3_ = 204
Formula	Ba_10_Fe_72_Zn_8_O_126_
Crystal system	Trigonal
Space group	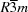
*a* = *b* (Å)	5.8721 (1)
*c* (Å)	487.184 (2)
*V* (Å^3^)	14548.23 (5)
*Z*	6
Sequence	MYMY_2_MYMY_4_
ρ_calc_ (Mg m^−3^)	5.401
*T* (K)	100
μ (mm^−1^)	14.111
Shape, colour	Platelet, black
Size (µm^3^)	69×48×31
λ (Å)	0.6889
*R*_1_	0.0735
Goodness-of-fit	1.090

**Table 7:** Site potentials for each of the symmetry-related tetrahedral groups in the (17)_2_ hexaferrite Each group is identified by its colour from Fig. 12 ▸(*a*) and site label in the CIF of the refined structure. The potentials have been averaged over the four tetrahedral sites in each group. In each column, the Fe layer with the least negative site potential is highlighted in bold.

Layering type	Site potential (V)	
	0 Zn^2+^ groups, Fe^2.9+^	1 Zn^2+^ group, Fe^2.95+^
TST (green – Fe)	**−30.004**	−24.951
T (blue – Fe07)	−31.077	−32.900
T (purple – Fe0A)	−31.876	−32.195
RST (red – Fe05)	−32.514	**−31.618**
RST (yellow – Fe06)	−33.184	−32.002

**Table 8:** Site potentials for each of the symmetry-related tetrahedral groups in the (34)_3_ hexaferrite Each group is identified by its colour from Fig. 12 ▸(*b*) and site label in the CIF of the refined structure. The potentials have been averaged over the six tetrahedral sites in each group. In each column, the Fe layer with the least negative site potential is highlighted in bold.

Layering type	Site potential (V)			
	0 Zn^2+^ groups, Fe^2.9+^	1 Zn^2+^ group, Fe^2.92+^	2 Zn^2+^ groups, Fe^2.95+^	3 Zn^2+^ groups, Fe^2.97+^
TST (light green – Fe1)	**−28.453**	−24.120	−27.573	−24.437
T (dark blue – Fe0E)	−28.630	−32.470	−36.290	−33.245
TST (dark green – Fe1A)	−28.839	**−31.458**	−27.080	−24.169
T (light blue – Fe0J)	−30.740	−31.807	−34.274	−32.151
T (light purple – Fe0Q)	−31.975	−31.880	−32.784	−31.394
RST (red – Fe08)	−32.913	−31.978	−31.280	**−30.995**
RST (orange – Fe0I)	−33.592	−31.978	−29.371	−33.388
T (dark purple – Fe0O)	−33.737	−31.833	**−29.146**	−26.646
RST (yellow – Fe0S)	−33.913	−32.543	−30.179	−32.876
RST (gold – Fe0B)	−34.065	−32.543	−31.167	−31.766

**Table 9:** Zinc force-field parameters from the work by Lewis *et al.* (1985 ▸)

A (eV)	ρ (Å)
700.3	0.3372
499.6	0.3595

## References

[bb1] Allan, D. R., Nowell, H., Barnett, S., Warren, M., Wilcox, A., Christensen, J., Saunders, L., Peach, A., Hooper, M., Zaja, L., Patel, S., Cahill, L., Marshall, R., Trimnell, S., Foster, A., Bates, T., Lay, S., Williams, M., Hathaway, P., Winter, G., Gerstel, M. & Wooley, R. (2017). *Crystals*, **7**, 336.

[bb2] Anderson, J. S. & Hutchison, J. L. (1975). *Contemp. Phys.* **16**, 443–467.

[bb3] Batlle, X., Obradors, X., Rodríguez–Carvajal, J., Pernet, M., Cabañas, M. V. & Vallet, M. (1991). *J. Appl. Phys.* **70**, 1614–1623.

[bb4] Blake, R. L., Hessevick, R. E., Zoltai, T. & Finger, L. W. (1966). *Am. Mineral.* **51**, 123–129.

[bb59] Brandenbry, K. (2006). *DIAMOND*. Crystal Impact GbR, Bonn, Germany.

[bb5] Braun, P. B. (1957). *Philips Res. Rep.* **12**, 491–548.

[bb6] Brixner, L. K. (1959). *J. Am. Chem. Soc.* **81**, 3841–3843.

[bb7] Collomb, A., Muller, J., Guitel, J. C. & Desvignes, J. M. (1989). *J. Magn. Magn. Mater.* **78**, 77–84.

[bb8] Cook, C. F. (1967). *J. Appl. Phys.* **38**, 2488–2496.

[bb9] Cook, C. F. & Nye, W. F. (1967). *Mater. Res. Bull.* **2**, 1–12.

[bb10] Dolomanov, O. V., Bourhis, L. J., Gildea, R. J., Howard, J. A. K. & Puschmann, H. (2009). *J. Appl. Cryst.* **42**, 339–341.

[bb11] Evans, P. (2006). *Acta Cryst.* D**62**, 72–82. 10.1107/S0907444905036693 16369096

[bb12] Evans, P. R. & Murshudov, G. N. (2013). *Acta Cryst.* D**69**, 1204–1214. 10.1107/S0907444913000061 PMC3689523 23793146

[bb13] Feuerbacher, M. *et al.* (2007). *Z. Kristallogr.* **222**, 259–288.

[bb14] Fredrickson, D. C., Lee, S. & Hoffmann, R. (2007). *Angew. Chem. Int. Ed.* **46**, 1958–1976. 10.1002/anie.200601678 17286326

[bb15] Gale, J. D. & Rohl, A. L. (2003). *Mol. Simul.* **29**, 291–341.

[bb16] Gambino, R. J. & Leonhard, F. (1961). *J. Am. Ceram. Soc.* **44**, 5, 221–224.

[bb17] Hopfinger, Th., Shcherban, O. O., Galez, Ph., Gladyshevkii, R. E., Lomello-Tafin, M., Jorda, J. L. & Couach, M. (2002). *J. Alloys Compd.* **333**, 237–248.

[bb18] Kohn, J. A. & Eckart, D. W. (1963). *J. Phys. Chem.* **67**, 957–958.

[bb19] Kohn, J. A. & Eckart, D. W. (1964). *Z. Kristallogr.* **119**, 454–464.

[bb20] Kohn, J. A. & Eckart, D. W. (1965*a*). *Am. Mineral.* **50**, 1371–1380.

[bb21] Kohn, J. A. & Eckart, D. W. (1965*b*). *J. Appl. Phys.* **36**, 1171–1172.

[bb22] Kohn, J. A. & Eckart, D. W. (1967). *Z. Kristallogr.* **124**, 69–76.

[bb23] Kohn, J. A., Eckart, D. W. & Cook, C. F. (1967). *Mater. Res. Bull.* **2**, 55–68.

[bb24] Kohn, J. A., Eckart, D. W. & Cook, C. F. (1971). *Science*, **172**, 519–525. 10.1126/science.172.3983.519 17802207

[bb25] Lewis, G. V. & Catlow, C. R. A. (1985). *J. Phys. C. Solid State Phys.* **18**, 1149–1161.

[bb26] Lim, J. T. & Kim, C. S. (2014). *J. Appl. Phys.* **115**, 17D706.

[bb27] Lim, J. T. & Kim, C. S. (2015). *J. Appl. Phys.* **117**, 17B743.

[bb28] Lim, J. T., Shim, I., Kim, C. S. & Sur, J. C. (2017). *J. Korean Phys. Soc.* **70**, 81–84.

[bb29] Lisjak, D. & Drofenik, M. (2004). *J. Magn. Magn. Mater.* **272–276**, E1817–E1819.

[bb30] Liu, X., Hernández-Gómez, P., Huang, K., Zhou, S., Wang, Y., Cai, X., Sun, H. & Ma, B. (2006). *J. Magn. Magn. Mater.* **305**, 524–528.

[bb31] Maglia, F., Buscaglia, V., Gennari, S., Ghigna, P., Dapiaggi, M., Speghini, A. & Bettinelli, M. (2006). *J. Phys. Chem. B*, **110**, 6561–6568. 10.1021/jp055713o 16570955

[bb32] Mardix, S. (1986). *Phys. Rev. B*, **33**, 8677–8684. 10.1103/physrevb.33.8677 9938269

[bb33] McConnell, J. D. M., Hutchison, J. L. & Anderson, J. S. (1974). *Proc. R. Soc. London Ser. A*, **339**, 1–12.

[bb34] Obulesu, K. R., Rao, T. S. & Raju, K. C. J. (2017). *J. Alloy Compd.* **695**, 3030–3035.

[bb35] Odeh, I., El Ghanem, H. M., Mahmood, S. H., Azzam, S., Bsoul, I. & Lehlooh, A.-F. (2016). *Physica B*, **494**, 33–40.

[bb36] Özgür, Ü., Ümit, Alivov, Y. & Morkoç, H. (2009). *J. Mater. Sci. Mater. Electron.* **20**, 789–834.

[bb37] Pałosz, B. (1983). *Acta Cryst.* C**39**, 521–528.

[bb38] Pullar, R. C. (2012). *Prog. Mater. Sci.* **57**, 1191–1334.

[bb60] Rigaku (2009). *CrystalClear.* Rigaku Corporation, Tokyo, Japan.

[bb39] Santoro, A. & Mighell, A. D. (1970). *Acta Cryst.* A**26**, 124–127.

[bb40] Savage, R. O. & Tauber, A. (1964). *J. Am. Ceram. Soc.* **47**, 13–18.

[bb41] Savage, R. O. & Tauber, A. (1967). *Mater. Res. Bull.* **2**, 469–478.

[bb44] Sheldrick, G. M., (2008*a*) *SADABS.* University of Göttingen, Germany.

[bb42] Sheldrick, G. M. (2008*b*). *Acta Cryst.* A**64**, 112–122. 10.1107/S0108767307043930 18156677

[bb43] Sheldrick, G. M. (2015). *Acta Cryst.* A**71**, 3–8.

[bb45] Siegrist, T., Svensson, C., Vanderah, T. A. & Roth, R. S. (2000). *Solid State Sci.* **2**, 539–544.

[bb46] Siegrist, T., Vanderah, T. A., Svensson, C. & Roth, R. S. (2002). *Solid State Sci.* **4**, 911–916.

[bb47] Siegrist, T., Vanderahb, T. A., Ramirez, A. P., Geyer, R. G. & Roth, R. S. (1998). *J. Alloys Compd.* **274**, 169–178.

[bb48] Soda, M., Ishikura, T., Nakamura, H., Wakabayashi, Y. & Kimura, T. (2011). *Phys. Rev. Lett.* **106**, 087201. 10.1103/PhysRevLett.106.087201 21405595

[bb49] Stergiou, C. A. & Litsardakis, G. (2016). *J. Magn. Magn. Mater.* **405**, 54–61.

[bb50] Togo, A. (2009). *Spglib*. https://atztogo.github.io/spglib/.

[bb51] Turner, G., Stewart, B., Baird, T., Peacock, R. D. & Cairns-Smith, A. G. (1996). *J. Cryst. Growth*, **158**, 276–283.

[bb52] Van Landuyt, J., Amelinckx, S., Kohn, J. A. & Eckart, D. W. (1974). *J. Solid State Chem.* **9**, 103–119.

[bb53] Verma, A. R. & Krishna, P. (1966). *Polymorphism and Polytypism in Crystals.* New York: Wiley.

[bb54] Villars, P. & Cenzual, K. (2017/2018). *Pearson’s Crystal Data: Crystal Structure Database for Inorganic Compounds*, Release 2017/18. ASM International, Materials Park, Ohio, USA.

[bb55] Weber, T., Dshemuchadse, J., Kobas, M., Conrad, M., Harbrecht, B. & Steurer, W. (2009). *Acta Cryst.* B**65**, 308–317. 10.1107/S0108768109014001 19461140

[bb56] Winn, M. D., Ballard, C. C., Cowtan, K. D., Dodson, E. J., Emsley, P., Evans, P. R., Keegan, R. M., Krissinel, E. B., Leslie, A. G. W., McCoy, A., McNicholas, S. J., Murshudov, G. N., Pannu, N. S., Potterton, E. A., Powell, H. R., Read, R. J., Vagin, A. & Wilson, K. S. (2011). *Acta Cryst.* D**67**, 235–242. 10.1107/S0907444910045749 PMC3069738 21460441

[bb57] Winter, G. (2010). *J. Appl. Cryst.* **43**, 186–190.

[bb58] Woodley, S. M., Battle, P. D., Gale, J. D., Richard, A. & Catlow, C. (1999). *Phys. Chem. Chem. Phys.* **1**, 2535–2542.

