# Supplementary material for: Structure determination and crystal chemistry of large repeat mixed-layer hexaferrites
Source: IUCrJ. 2018 Sep 12;5(Pt 6):681–98. doi: 10.1107/S2052252518011351 (PMC6211530; doi:10.1107/S2052252518011351)
Supplement: Supplementary file 2 [file m-05-00681-sup2.pdf]

IUCrJ

Volume 5 (2018)

Supporting information for article:

Structure determination and crystal chemistry of large repeat mixed-layer hexaferrites

C. Delacotte, G. F. S. Whitehead, M. J. Pitcher, C. M. Robertson, P. M. Sharp, M. S. Dyer, J. Alaria, J. B. Claridge, G. R. Darling, D. R. Allan, Graeme Winter and M. J. Rosseinsky

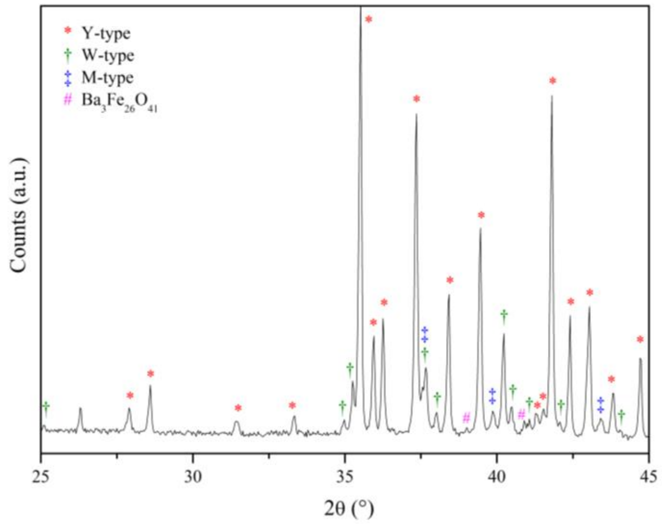

Figure S1: Representative PXRD pattern of the ceramic syntheses at the nominal composition $Ba_{10}Fe_{72}Zn_8O_{126}$, fired at 1300 °C for 24 hours. Symbols are assigned to the different phases present in the pattern.

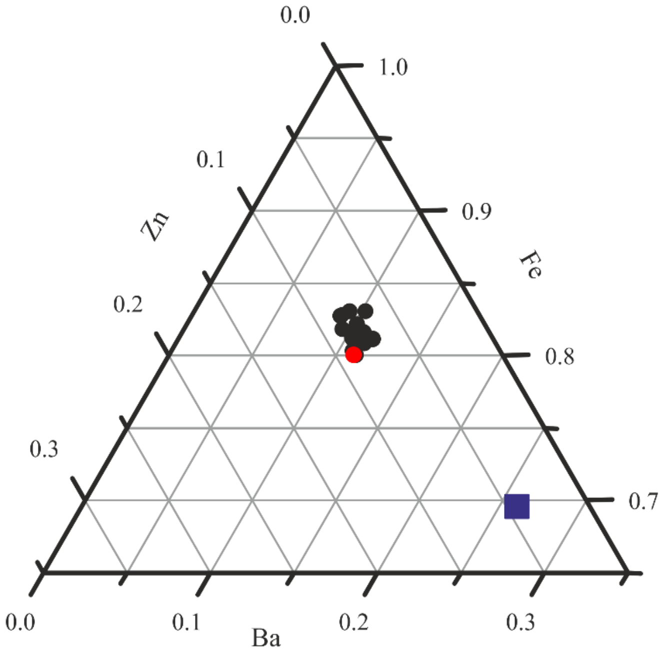

Figure S2: Ba-Fe-Zn ternary diagram. Black points correspond to experimental EDX measurements performed on the $(17)_2$, $(34)_3$ and $(68)_3$ hexaferrites, the red point to the refined composition from Xray- structure analysis, and the blue square the starting composition of the best crystal growth batch.

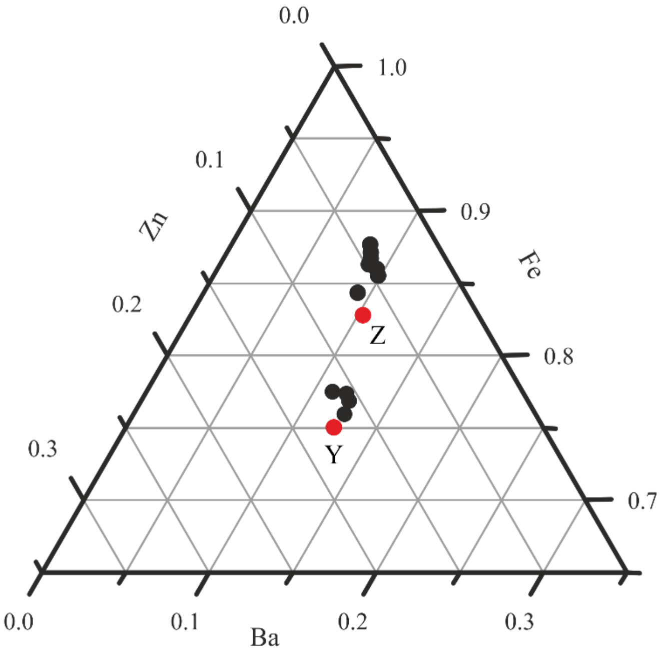

Figure S3. Black points relate experimental EDX measurements performed on crystals of Z and Y-type hexaferrites and red points the corresponding ideal compositions.

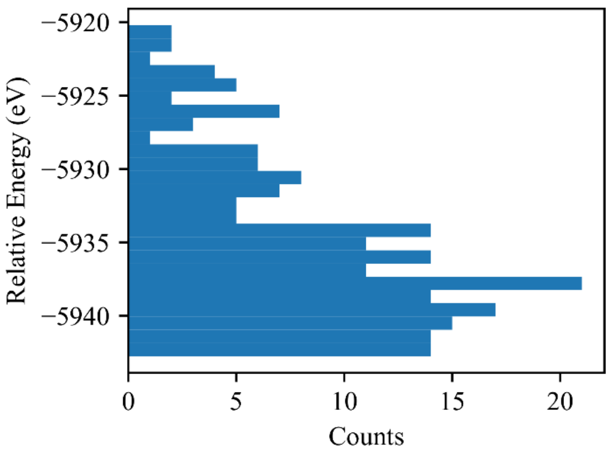

Figure S4: The energy of each of the 210 structures generated by populating Zn atoms in different symmetry related groups of tetrahedral sites for the $(34)_3$ hexaferrite, relative to the lowest energy structure.

Table S1 Niggli reduced cells of the nine hexaferrites with refined crystal structures reported in this paper. Cell reduction was performed using the python implementation of Spglib (Togo, 2009).

Hexaferrite	a (Å)	b (Å)	c (Å)	α (°)	β (°)	γ (°)	Volume (Å^3)
$(17)_2$	5.874	5.874	81.239	90.000	90.000	120.000	2427.160
$(40)_1$	5.872	5.872	95.725	90.000	90.000	120.000	2858.541
$(28)_3$	5.874	5.874	66.798	87.480	87.480	60.000	1993.142
$(34)_3$	5.870	5.870	81.269	87.930	87.930	60.000	2423.311
$(40)_3$	5.872	5.872	95.789	88.244	88.244	60.000	2858.251
$(52)_3$	5.870	5.870	124.771	88.652	88.652	60.000	3721.895
$(58)_3$	5.868	5.868	139.256	88.793	88.793	60.000	4151.353
$(64)_3$	5.868	5.868	153.779	88.907	88.907	60.000	4584.051
$(68)_3$	5.872	5.872	162.430	88.964	88.964	60.000	4849.427

Table S2: Details of the single crystal structure refinement for the $(28)_3$ hexaferrite

Series	M_2Y_3
Number of anion layers	$(28)_3$=84
Formula	$Ba_8Fe_{60}Zn_6O_{104}$
Crystal system	Trigonal
Space group	$R\bar{3}m$
a=b (Å)	5.87358(4)
c (Å)	200.135(3)
V (Å^3)	5979.4(1)
Z	3
Sequence	$MYMY_2$
$\rho_{calc.}$ (g/cm^3)	5.399
T (K)	100
μ (mm^{-1})	14.149
Shape and colour	Platelet, black
Size (μm^3)	64×50×12
λ (Å)	0.6889
R_1	0.0411
G.O.F.	1.094

Table S3: Details of the single crystal structure refinement for the $(17)_2$ hexaferrite

Series	M_2Y_4
Number of anion layers	34
Formula	$Ba_{10}Fe_{72}Zn_8O_{126}$
Crystal system	Hexagonal
Space group	$P6_3/mmc$
a=b (Å)	5.87357(4)
c (Å)	81.2388(8)
V (Å^3)	2427.16(4)
Z	1
Sequence	MY_2MY_2
$\rho_{calc.}$ (g/cm^3)	5.397
T (K)	100
μ (mm^{-1})	14.737
Shape and colour	Platelet, black
Size (μm^3)	64×53×20
λ (Å)	0.6889
R_1	0.0387
G.O.F.	1.119

Table S4: Details of the single crystal structure refinement for the $(40)_1$ hexaferrite

Series	M_2Y_5
Number of anion layers	(40)
Formula	$Ba_6Fe_{42}Zn_5O_{74}$
Crystal system	Trigonal
Space group	$P\bar{3}m1$
a=b (Å)	5.87212(3)
c (Å)	95.725(1)
V (Å^3)	2858.54(4)
Z	2
Sequence	$MYMY_4$
$\rho_{calc.}$ (g/cm^3)	5.411
T (K)	100
μ (mm^{-1})	14.263
Shape and colour	Platelet, black
Size (μm^3)	59×38×21
λ (Å)	0.6889
R_1	0.0545
G.O.F.	1.072

Table S5: Details of the single crystal structure refinement for the $(40)_3$ hexaferrite

Series	M_2Y_5
Number of anion layers	$(40)_3$=120
Formula	$Ba_6Fe_{42}Zn_5O_{74}$
Crystal system	Trigonal
Space group	$R\bar{3}m$
a=b (Å)	5.87169(8)
c (Å)	287.187(7)
V (Å^3)	8574.7(3)
Z	6
Sequence	MY_2MY_3
$\rho_{calc.}$ (g/cm^3)	5.412
T (K)	100
μ (mm^{-1})	14.263
Shape and colour	Platelet, black
Size (μm^3)	64×37×24
λ (Å)	0.6889
R_1	0.0564
G.O.F.	1.122

Table S6: Details of the single crystal structure refinement for the $(52)_3$ hexaferrite

Series	M_2Y_7
Number of anion layers	$(52)_3$=156
Formula	$Ba_8Fe_{54}Zn_7O_{96}$
Crystal system	Trigonal
Space group	$R\bar{3}m$
a=b (Å)	5.870(1)
c (Å)	374.176(1)
V (Å^3)	11165.61(4)
Z	6
Sequence	$MYMY_6$
$\rho_{calc.}$ (g/cm^3)	5.425
T (K)	100
μ (mm^{-1})	15.115
Shape and colour	Platelet, black
Size (μm^3)	74×29×18
λ (Å)	0.6889
R_1	0.0308
G.O.F.	1.082

Table S7: Details of the single crystal structure refinement for the $(58)_3$ hexaferrite

Series	M_2Y_8
Number of anion layers	$(58)_3$=174
Formula	$Ba_9Fe_{60}Zn_8O_{107}$
Crystal system	Trigonal
Space group	$R\bar{3}m$
a=b (Å)	5.868(1)
c (Å)	417.644(1)
V (Å^3)	12454.23(3)
Z	6
Sequence	MY_3MY_5
$\rho_{calc.}$ (g/cm^3)	5.430
T (K)	100
μ (mm^{-1})	14.403
Shape and colour	Platelet, black
Size (μm^3)	96×76×16
λ (Å)	0.6889
R_1	0.0337
G.O.F.	1.103

Table S8: Details of the single crystal structure refinement for the $(64)_3$ hexaferrite

Series	M_2Y_9
Number of anion layers	$(64)_3$=192
Formula	$Ba_{10}Fe_{66}Zn_9O_{118}$
Crystal system	Trigonal
Space group	$R\bar{3}m$
a=b (Å)	5.8676(1)
c (Å)	461.224(1)
V (Å^3)	13751.92(3)
Z	6
Sequence	MY_4MY_5
$\rho_{calc.}$ (g/cm^3)	5.429
T (K)	100
μ (mm^{-1})	14.392
Shape and colour	Platelet, black
Size (μm^3)	135×124×30
λ (Å)	0.6889
R_1	0.0478
G.O.F.	1.084

Table S9: Refined Fe-O distances for the $(34)_3$ hexaferrite

Fe label	O label	Distance (Å)	Multiplicity
Fe08	O01B	1.912(6)	1
Fe08	O01E	1.920(4)	3
Fe09	O01E	2.020(4)	3
Fe09	O01Q	1.988(4)	3
Fe0A	O01L	2.010(4)	3
Fe0A	O01P	2.007(4)	3
Fe0B	O019	1.971(7)	1
Fe0B	O01Q	1.888(4)	3
Fe0C	O014	2.036(4)	3
Fe0C	O01I	2.024(4)	3
Fe0D	O012	2.079(3)	3
Fe0D	O01J	1.962(4)	3
Fe0E	O01F	1.874(7)	1
Fe0E	O01G	1.893(4)	3
Fe0F	O01G	2.026(4)	6
Fe0G	O012	2.071(3)	3
Fe0G	O01H	1.967(3)	3
Fe0H	O010	2.010(3)	1
Fe0H	O015	2.009(4)	1
Fe0H	O01C	1.975(3)	2
Fe0H	O01L	2.050(2)	2
Fe0I	O01R	1.921(4)	3
Fe0I	O1	1.907(6)	1
Fe0J	O010	1.881(7)	1
Fe0J	O014	1.888(4)	3
Fe0K	O01G	2.260(4)	3
Fe0K	O01K	1.929(4)	3
Fe0L	O017	1.976(3)	1
Fe0L	O01B	2.065(4)	1
Fe0L	O01J	1.929(2)	2
Fe0L	O01Q	2.110(2)	2
Fe0M	O01F	2.015(4)	1

Fe0M	O01K	1.973(2)	2
Fe0M	O01M	2.011(4)	1
Fe0M	O01P	2.051(2)	2
Fe0N	O014	2.253(4)	3
Fe0N	O01C	1.930(4)	3
Fe0O	O00Z	1.877(6)	1
Fe0O	O01N	1.893(4)	3
Fe0P	O011	1.971(2)	2
Fe0P	O013	2.006(4)	1
Fe0P	O019	2.025(4)	1
Fe0P	O01E	2.062(2)	2
Fe0Q	O013	1.882(7)	1
Fe0Q	O01I	1.896(4)	3
Fe0R	O011	1.934(4)	3
Fe0R	O01I	2.247(4)	3
Fe0S	O018	1.968(7)	1
Fe0S	O01A	1.890(4)	3
Fe0T	O016	1.967(3)	1
Fe0T	O01A	2.113(2)	2
Fe0T	O01H	1.924(2)	2
Fe0T	O1	2.069(4)	1
Fe0U	O01A	1.983(3)	3
Fe0U	O01R	2.024(4)	3
Fe0V	O01N	2.229(4)	3
Fe0V	O01O	1.938(3)	3
Fe0W	O01N	2.035(4)	6
Fe0X	O00Z	2.009(3)	1
Fe0X	O018	2.023(4)	1
Fe0X	O01O	1.970(2)	2
Fe0X	O01R	2.061(2)	2
Fe0Y	O012	1.841(3)	3
Fe0Y	O016	2.329(7)	1
Fe0Y	O017	2.317(7)	1

Table S10: Relative energies (eV) of the structures in Figure 13 with different force-field parameters.

Zn Blocks		Original Parameter Set	Parameter Set: A=700.3eV, ρ = 0.3372Å	Parameter Set: A = 499.6eV, ρ = 0.3595Å
T	T	0.000	0.000	0.000
T	RST	0.050	0.255	0.624
RST	TST	0.632	0.904	1.648
RST	T	0.887	1.001	1.372
RST	TST	2.613	2.833	3.420
RST	T	2.861	2.932	3.151
T	TST	4.701	4.895	5.251
RST	T	5.677	5.610	5.442
RST	T	7.221	7.317	7.582
RST	RST	16.24	16.71	17.02
